# Optimising T cell (re)boosting strategies for adenoviral and modified vaccinia Ankara vaccine regimens in humans

**DOI:** 10.1038/s41541-020-00240-0

**Published:** 2020-10-12

**Authors:** Stefania Capone, Anthony Brown, Felicity Hartnell, Mariarosaria Del Sorbo, Cinzia Traboni, Ventzislav Vassilev, Stefano Colloca, Alfredo Nicosia, Riccardo Cortese, Antonella Folgori, Paul Klenerman, Eleanor Barnes, Leo Swadling

**Affiliations:** 1 ReiThera Srl, Via di Castel Romano, 100, 00128 Rome, Italy; 2 Nuffield Department of Medicine, University of Oxford, Oxford, UK; 3 GSK Vaccines, Brussels, Belgium; 4 Keires AG, Baumleingasse 18, CH 4051 Basel, Switzerland; 5 CEINGE, via Gaetano Salvatore 486, 80145 Naples, Italy; 6 Department of Molecular Medicine and Medical Biotechnology, University of Naples Federico II, Via S. Pansini 5, 80131 Naples, Italy; 7 Oxford NIHR BRC, and Translational Gastroenterology Unit, Oxford, UK; 8 The Jenner Institute, University of Oxford, Oxford, UK; 9 Present Address: Nouscom Srl, Via di Castel Romano, 100, 00128 Rome, Italy; 10 Present Address: Rayne Institute, University College London, London, UK

**Keywords:** Live attenuated vaccines, Adaptive immunity, Vaccines

## Abstract

Simian adenoviral and modified vaccinia Ankara (MVA) viral vectors used in heterologous prime-boost strategies are potent inducers of T cells against encoded antigens and are in advanced testing as vaccine carriers for a wide range of infectious agents and cancers. It is unclear if these responses can be further enhanced or sustained with reboosting strategies. Furthermore, despite the challenges involved in MVA manufacture dose de-escalation has not been performed in humans. In this study, healthy volunteers received chimpanzee-derived adenovirus-3 and MVA vaccines encoding the non-structural region of hepatitis C virus (ChAd3-NSmut/MVA-NSmut) 8 weeks apart. Volunteers were then reboosted with a second round of ChAd3-NSmut/MVA-NSmut or MVA-NSmut vaccines 8 weeks or 1-year later. We also determined the capacity of reduced doses of MVA-NSmut to boost ChAd3-NSmut primed T cells. Reboosting was safe, with no enhanced reactogenicity. Reboosting after an 8-week interval led to minimal re-expansion of transgene-specific T cells. However, after a longer interval, T cell responses expanded efficiently and memory responses were enhanced. The 8-week interval regimen induced a higher percentage of terminally differentiated and effector memory T cells. Reboosting with MVA-NSmut alone was as effective as with ChAd3-NSmut/MVA-NSmut. A ten-fold lower dose of MVA (2 × 10^7^pfu) induced high-magnitude, sustained, broad, and functional Hepatitis C virus (HCV)-specific T cell responses, equivalent to standard doses (2 × 10^8^ pfu). Overall, we show that following Ad/MVA prime-boost vaccination reboosting is most effective after a prolonged interval and is productive with MVA alone. Importantly, we also show that a ten-fold lower dose of MVA is as potent in humans as the standard dose.

## Introduction

Diverse vaccine modalities have been tested in the past in an attempt to recapitulate the T cell mediated life-long immunity that can be induced upon acute-resolving viral infection. Great advances in the field of molecular virology have enabled the manipulation of viruses as vectors to deliver foreign antigens and these have emerged as the most potent method of inducing an antigen-specific T cell response (reviewed in refs. ^1,2^). These viruses have highly evolved mechanisms of cell entry and protein expression that can be exploited for the delivery of selected immunogenic antigens, whilst serial passage (in the case of modified vaccinia Ankara^3^) or gene deletions (for adenoviruses) can render these viral vectors non-pathogenic and non-replicative.

The best characterized viral vectors and the most widely used in current phase I and II clinical trials are Adenoviral (Ad) vectors and MVA (World Health Organisation International Clinical Trials Registry, http://apps.who.int/trialsearch^1^). Ad^4,5^ and MVA^6–8^ vectors are being developed as candidate vaccines for a diverse range of pathogens, including several prime-boost regimens making use of Ad vector’s propensity to prime a strong T cell response (MERS^9^; SARS-Cov-2^10^), and MVA’s ability to broaden and enhance both peak and memory transgene-specific T cell responses (HCV^11^; Influenza^12^; Ebola^13^; Malaria^8^**;** RSV^14^**;** HIV^15,16^). Ad/MVA prime-boost regimens are not only highly immunogenic, but have shown encouraging efficacy in animal and human challenge models (Ebola^17^, Malaria^18^, SIV^19^, RSV^20^). Although Ad and MVA vectors used in prime-boost strategies are both potent and safe, few studies have investigated whether reboosting strategies may be employed to further enhance immunogenicity, whilst also retaining their safety and minimal reactogenicity^6,21,22^.

We have developed a T cell-based vaccine regimen for Hepatitis C virus (HCV), employing a chimpanzee-derived adenovirus 3 (ChAd3) prime and an MVA boost encoding the non-structural region of HCV (NSmut; genotype 1b), which was the first vaccine for HCV to progress to Phase II efficacy testing (NCT01436357). Although, we have shown these vaccines to be highly immunogenic, inducing high-magnitude polyfunctional CD4^+^ and CD8^+^ T cells^11^, recent analysis (NCT01436357) showed that this regimen was not effective in preventing chronic infection in intravenous drug using populations. Therefore, new strategies are required to enhance and/or sustain antiviral immunity.

In this study, we describe the results of a phase I clinical trial in which we investigated the impact of reboosting and dose de-escalation on the magnitude and quality of the T cell response induced in humans, using our candidate HCV vaccine as a model vaccine. Specifically, we assess the impact of a second round of ChAd3 and MVA vaccinations, or MVA vaccination alone, given after a short or long interval following initial ChAd3/MVA prime-boost.

Additionally, since MVA vaccine manufacture is challenging and using effective lower doses could increase the number of vials that are produced in this process, so benefiting large-scale vaccine programs^23^, we perform a comprehensive analysis of the HCV-specific T cells response induced by lower doses of MVA boost vaccination.

## Results

### Reboosting vaccinations with ChAd3-NSmut and MVA-NSmut are safe and well tolerated

**Fig. 1:**
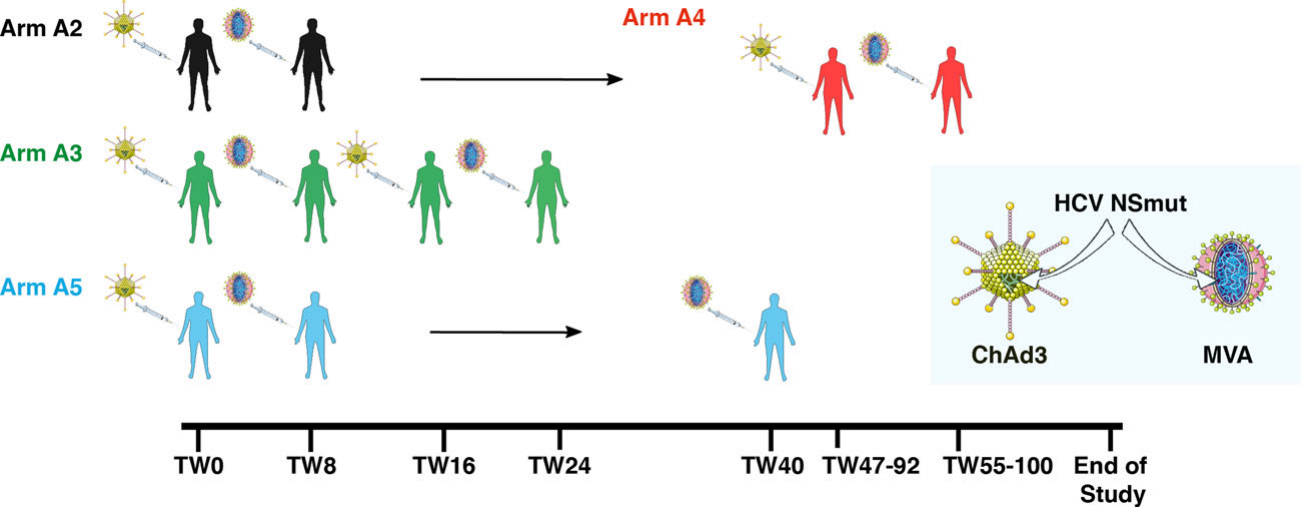
Reboosting vaccination schedules. A chimpanzee-derived adenovirus 3 (ChAd3) and a modified vaccinia Ankara (MVA) were engineered to express the non-structural region (NS3-5b, BK strain genotype 1b, NSmut) of hepatitis C virus. Healthy volunteers were given a prime-boost vaccine regimen (ChAd3-NSmut at trial week 0 [TW0], MVA-NSmut boost at TW8) followed by reboosting vaccination with a second round of ChAd3-NSmut/MVA-NSmut after a short interval (all vaccines 8 weeks apart, arm A3), after a long interval (TW47-92, arm A4; volunteers re-recruited from arm A2), or were reboosted with MVA-NSmut alone after a long interval (TW40, arm A5). Vaccine dose 2.5 × 10^10^ viral particles (vp) for ChAd3-NSmut and 2 × 10^8^ plaque forming units (pfu) for MVA-NSmut.

A primary endpoint for this phase-I clinical trial was to assess the safety and tolerability of secondary administration of Ad and MVA vectors in healthy volunteers. The volunteer arm protocols are described in Fig. 1 and Supplementary Table 1 and volunteer demographics in Supplementary Table 2. Following ChAd3-NSmut vaccination (whether first or second administrations) most adverse events (AEs; 97.5%) were mild or moderate and there were no serious adverse reactions (Supplementary Fig. 1 and Supplementary Table 3). As previously described^11,14^, AEs of all severities were more common after MVA-NSmut (chi-square *p* = 0.0468; Supplementary Table 3). Importantly, in volunteers who were re-vaccinated (arms A3-A5) with a second round of ChAd3 and/or MVA, reboosting vaccinations resulted in a similar AE profile to the first round of vaccinations regardless of time delay between vaccinations (8–92 weeks; Supplementary Fig. 1**;** Supplementary Table 3). Overall, both ChAd3-NSmut and MVA-NSmut were well tolerated regardless of the number of administrations, the order in which they were administered, or the time between vaccinations.

### The magnitude of the peak and memory vaccine-induced HCV-specific T cell response is improved when the interval before reboosting is prolonged

**Fig. 2:**
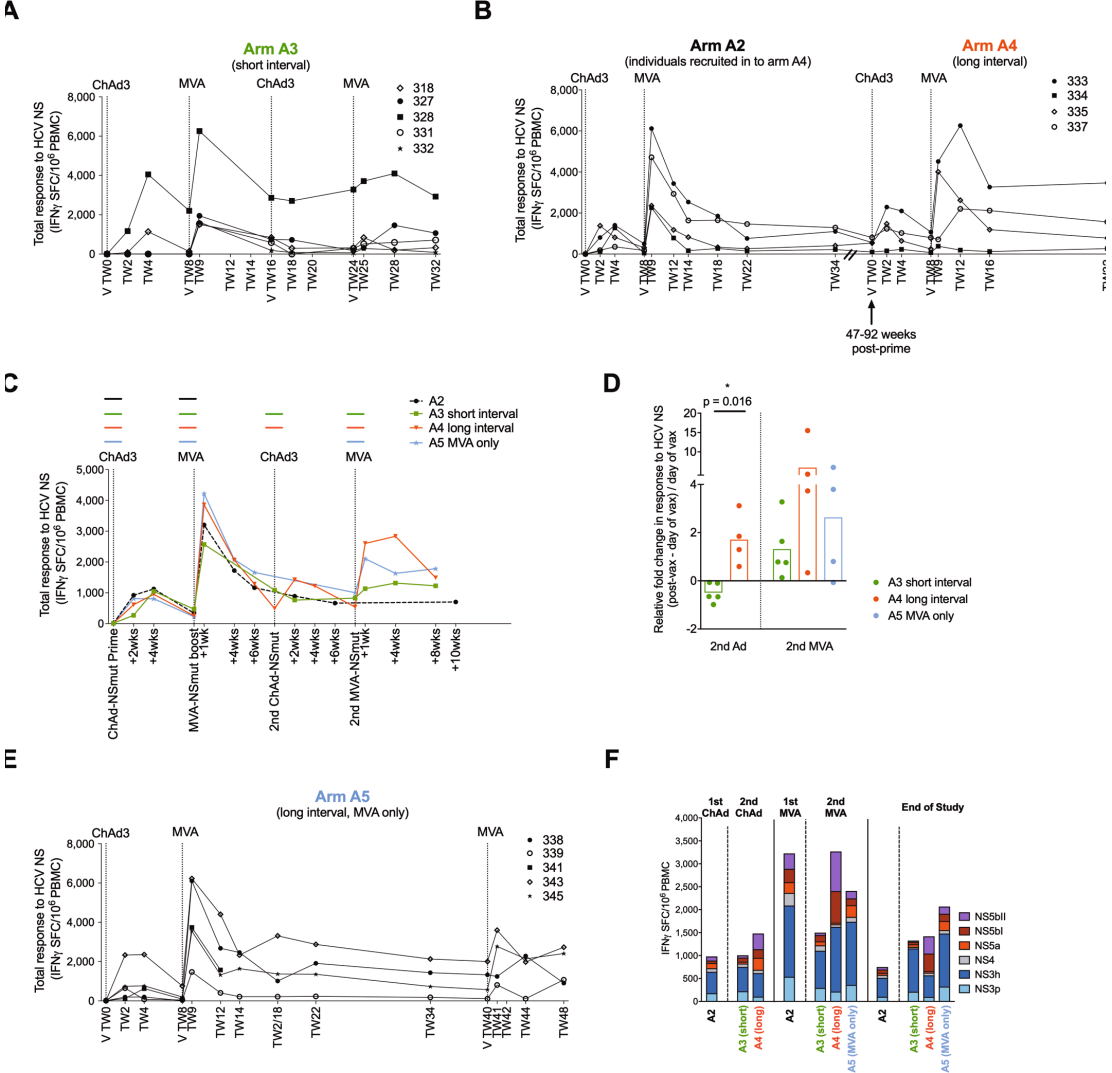
HCV-specific T cell responses are expanded by reboosting after a long interval with a second round of ChAd3-NSmut and MVA-NSmut or with MVA-NSmut alone. **a**–**c** The total ex vivo T cell response to the non-structural (NS) region of HCV encoded within the vaccine is shown over time (IFNγ ELISpot; spot forming cells per 10^6^ PBMC; calculated by summing the responses to positive peptide pools corrected for background; materials and methods). Individual healthy volunteers are shown for arms A3 (**a**), arm A2/A4 (**b**) and overlaid group means (**c**). Colored horizontal lines above the graph indicate when a vaccine was given. **d** Fold change in T cell response to HCV NS (post-vaccination response–pre-vaccination response/pre-vaccination response). Post-vaccination response was taken as +2 weeks for ChAd3, +1 week for MVA. Bars at group mean. **e** The total ex vivo T cell response to the NS region of HCV over time for individual volunteers for arm A5. **a**–**c**, **e** Vertical dashed lines indicate time of vaccination. chAd3, chimpanzee-derived adenovirus 3; MVA, modified vaccina Ankara. **f** The ex vivo T cell response to HCV by antigen at the peak of the T cell response post-vaccination and at the end of the study (2–4 weeks post-ChAd3-NSmut and 1–4 weeks post-MVA-NSmut for peak response and 14–40 weeks post vaccination for end of study; group mean per peptide pool by IFNγ ELISpot, *n* = 4–9; all pools background subtracted).

We assessed whether the response to vaccination could be enhanced in magnitude or functionality by administering a second cycle of ChAd3-NSmut/MVA-NSmut vaccination after a short (8 weeks- Arm A3) or long time interval (39–84 weeks- Arm A4; volunteers re-enrolled from initial clinical trial of ChAd3-NSmut/MVA-NSmut, see methods; Fig. 1). When all four vaccines were given 8 weeks apart (Fig. 2a**; Arm A3**) no expansion of HCV NSmut-specific T cells was seen after the second ChAd3-NSmut vaccination in all five volunteers and only a weak expansion was seen after the second MVA-NSmut; however, when there was a long interval of 39–84 weeks between the first and second rounds of ChAd3-NSmut/MVA-NSmut (**Arm A4;** Fig. 2b) HCV-specific T cell responses expanded in three of the four volunteers to levels comparable to those seen in the initial round of vaccinations. The relative fold change in HCV-specific T cell response after vaccination (2 weeks post-ChAd3-NSmut and 1 week post-MVA-NSmut) was on average higher when a longer interval was used (Fig. 2c), but only reached significance after reboosting with ChAd3-NSmut (Fig. 2d).

To investigate whether reboosting with MVA-NSmut alone would be as effective as reboosting with ChAd3/MVA, 5 volunteers in Arm A5 received a second MVA-NSmut 32 weeks after their first MVA-NSmut boost (Fig. 2e). The peak magnitude of the HCV-specific T cells response after the second MVA vaccination did not differ when it was preceded by a second ChAd3-NSmut vaccination or was given alone (Fig. 2d, e; peak post second MVA-NSmut vaccination **arm A4** median 3384 range 385–6260, **arm A5** median 2532 range 832–3599, *p* = 0.6857).

We next assessed whether reboosting impacted on the breadth of the T cell response and the immunodominance of different HCV antigens encoded within the vaccine. Vaccination-induced T cells targeting all HCV non-structural proteins encoded within the vaccines and the relative contribution of each HCV antigen was relatively unchanged from post-prime vaccination to boosting with MVA and subsequent reboosting with ChAd3-NSmut and MVA-NSmut (Fig. 2f, Supplementary Fig. 3).

**Fig. 3:**
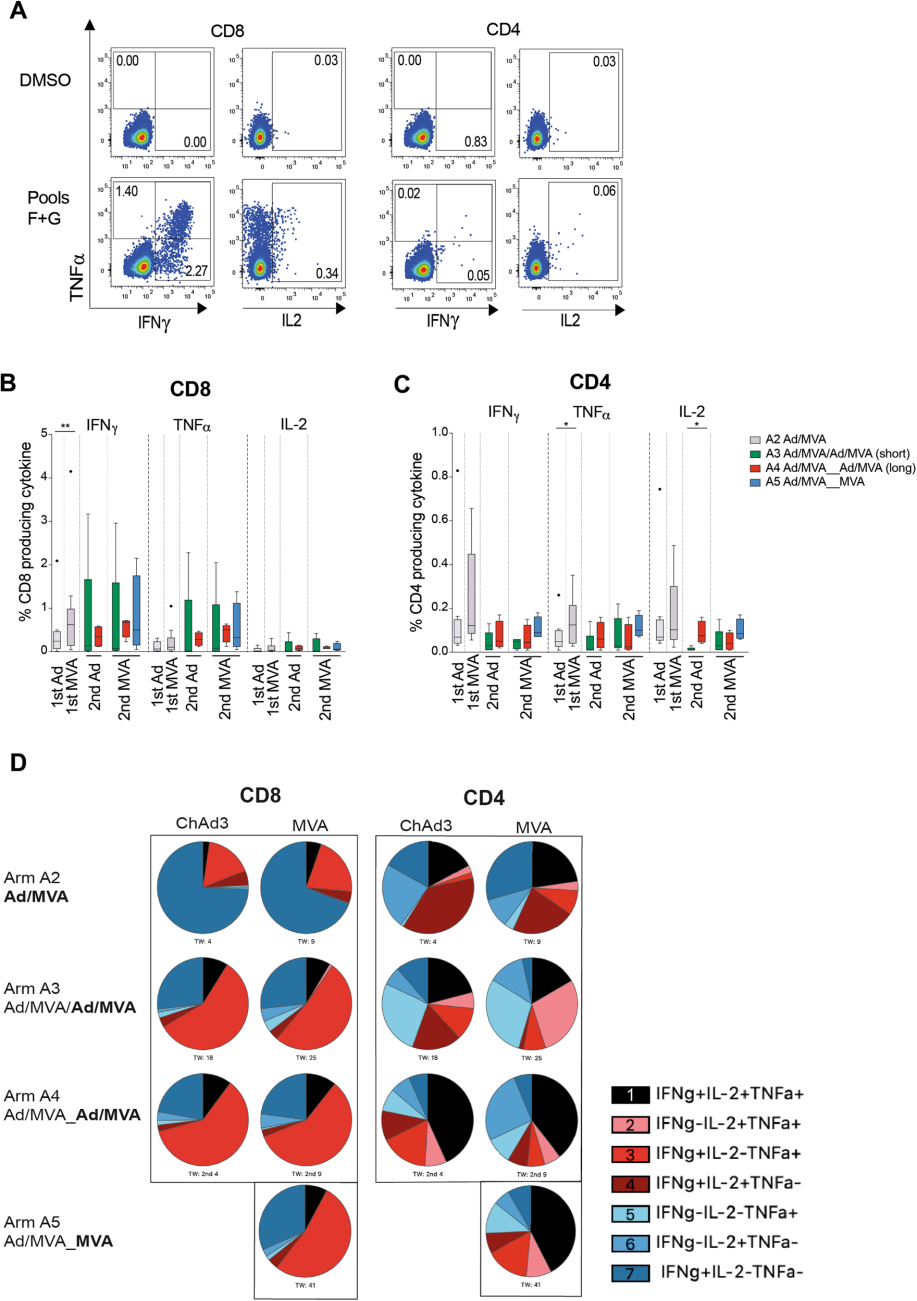
Cytokine-producing HCV-specific T cells are induced by reboosting after a long interval. **a**–**c** Comparison of ex vivo cytokine production by T cells at the time of peak magnitude of response (peak NS response by IFNγ ELISpot) to each vaccination (1–4 weeks post-ChAd3-NSmut and MVA-NSmut). **a** Example plots showing TNFα/IFNγ and IL-2/TNFα after ICS for CD4^+^ and CD8^+^ T cells stimulated with pools F+G or DMSO control (volunteer 343 arm A5 TW41, 2nd MVA; see Supplementary Fig. 4 for full gating strategy). The percentage of total CD8^+^ (**b**) or CD4^+^ (**c**) T cells producing IFNγ, TNFα, or IL-2 after overnight stimulation with peptides covering the non-structural region of HCV within the vaccines (Pools F+G, H+I, L+M summed after subtracting values for paired DMSO wells from each stimulation) are shown. **d** The proportion of the transgene-specific CD4^+^ or CD8^+^ T cells producing different combinations of IFNγ, TNFα, and IL-2 are presented as pie charts (base mean) at the peak of the T cell response post vaccination (samples with a transgene-specific response <0.025% of CD4^+^ or CD8^+^ T cells were excluded from polyfunctionality analysis; *n* = 4–9 per arm). **b**, **c** Box–whisker Tukey, outliers shown as dots (*n* = 9 A2, *n* = 5 arm A3, *n* = 4 arms A4, A5). Wilcoxon paired *t* test 1st Ad vs. 1st MVA, Mann–Whitney unpaired *t* test for 2nd Ad A3 vs. A4, Kruskal–Wallis one-way ANOVA with Dunn’s correction for 2nd MVA A3, A4, A5 per cytokine. Ad, adenovirus; MVA, modified vaccina Ankara. *P < 0.05; **P < 0.005.

The functionality of HCV-specific T cells induced by different vaccine regimens was tested at the time of peak magnitude after vaccination by ex vivo intracellular cytokines staining. For all cytokines tested (IFNγ, TNFα, and IL-2) the magnitude of the CD4^+^ and CD8^+^ cytokine-producing T cell response was larger when a longer interval was left between rounds of vaccination (**arm A4**: red > **arm A3**: green), in agreement with the IFNγ ELISpot data (Fig. 3). When reboosting after a long interval with a second MVA-NSmut vaccination T cell responses were comparable whether a second ChAd-NSmut was given before or not (**arm A4** red vs. **arm A5** blue). Overall CD4^+^ T cell responses were lower in magnitude than CD8^+^ T cell responses.

We next used boolean gating to assess whether the proportion of polyfunctional transgene-specific T cells, capable of producing IFNγ, TNFα, and IL-2 in combination, differ at the peak of the T cell response between different reboosting regimens. We have previously shown that polyfunctional T cells induced by ChAd3-NSmut/MVA-NSmut vaccination, that are capable of producing IFNγ, TNFα, and IL-2 in combination, produce a larger amount of each individual cytokine on a per cell basis, which may indicate greater antiviral activity^9^. As has been previously described, HCV NS-specific CD4^+^ T cells were more polyfunctional than CD8^+^ owing to their greater propensity to produce IL2 (Fig. 3d). After ChAd3-NSmut prime and MVA-NSmut boost the majority of transgene-specific CD8^+^ T cells were IFNγ single producers; however, a larger proportion were polyfunctional after reboosting regardless of the regimen used, in particular producing both IFNγ and TNFα. A larger population of CD4^+^ T cells producing all three cytokines was seen when reboosting with a further round of ChAd3-NSmut/MVA-NSmut (arm A4) or MVA-NSmut alone (arm A5) after a longer interval.

In summary, HCV-specific T cell responses were most effectively expanded and showed greatest polyfunctionality when individuals were re-vaccinated after an extended period of time, with a single second MVA vaccination as effective as re-vaccination with both Ad and MVA

### Monitoring anti-vector humoral and T cell responses

To evaluate the impact of pre-existing and vaccination-induced anti-ChAd3 vector immunity on the ability of volunteers to respond to repeated vaccine administration volunteers were monitored for ChAd3 neutralizing antibody (nAb) titers and for IFNγ secretion upon PBMC stimulation with a pool of overlapping peptides of the Ad5 Hexon protein sequence. Hexon is the most abundant Ad capsid protein and main target of T cell responses, with many described CD4^+^ and CD8^+^ epitopes residing in protein regions highly conserved across human and primate adenoviral strains^24–27^.

**Fig. 4:**
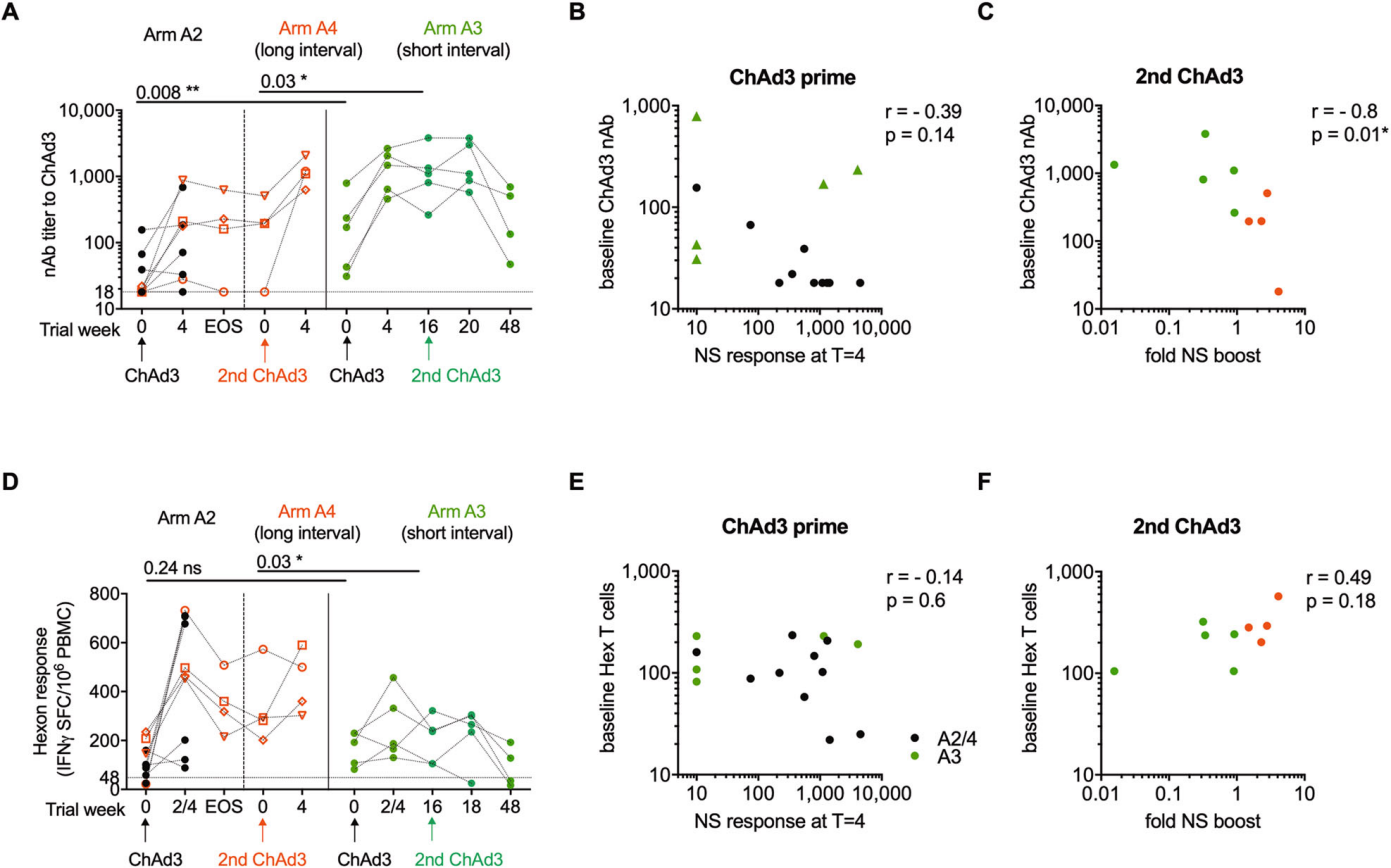
Anti-vector immunity. **a** Titer of neutralising antibodies (nAbs) against ChAd3 vector (dotted line at nAb titer of 18, limit of detection for the assay). Volunteers who received ChAd3-NSmut prime and MVA-NSmut boost as part of arm A2 which were then reboosted in arm A4 are indicated by red symbols. Trial week indicates weeks since first vaccination for arm A2 and A3 and time of second ChAd3 vaccination for Arm A4. **b** Anti-ChAd3 nAb titer at baseline vs. peak magnitude of T cell responses to NS region of HCV post-vaccination (IFNγ ELISpot; spot forming cells per 10^6^ PBMC). **c** Anti-ChAd3 nAb titer at time of second ChAd3-NSmut vaccination vs. fold change in magnitude of T cell responses to HCV NS post-vaccination. Fold change (FC) was calculated as (total NS response 2 weeks post-vaccination—NS response on day of second ChAd3-NSmut vaccination)/NS response pre-vaccination. **d** Ex vivo T cell response to Ad5 hexon (IFNγ ELISpot; horizontal dotted line at 48, positivity cut-off for the assay). Trial week indicates weeks since first vaccination for arm A2 and A3 and time of second ChAd3 vaccination for Arm A4. **e** T cell response to Ad5 hexon at baseline vs. peak magnitude of T cell responses to NS region of HCV post-vaccination. **f** T cell response to Ad5 hexon at time of second ChAd3-NSmut vaccination vs. fold change in magnitude of T cell responses to HCV NS post vaccination (FC calculated as above). **a**, **d** Paired Wilcoxon *t* test. **b**, **c**, **e**, **f** Spearman rank correlation. ChAd3, chimpanzee-derived adenovirus 3; EOS, end of study. NS, non-structural; MVA, modified vaccina Ankara.

ChAd3 was selected as the candidate adenoviral vector due to the low global prevalence of pre-existing nAb^28^; indeed this was found to be the case, with 12 of 14 individuals in groups A2 and A3 having a baseline ChAd3 cross-reacting nAb titer below 200 (Fig. 4a)—a titer previously associated with reduced immunogenicity of Ad5 vectored vaccines^29^. The magnitude of the HCV-specific T cell response 4 weeks (peak) after primary ChAd vaccination did not correlate with baseline ChAd3 nAb titers (Fig. 4b). Baseline nAb titers were overall higher in group A3 compared to group A2 (GeoMean [GM] 133 vs. 28, *p* = 0.008**), however, the vaccine-induced HCV-specific T cell response was not significantly different between arm A3 and A2 after ChAd priming (*p* = 0.38) or after the first MVA boost (*p* = 0.42; Fig. 2a). Overall, pre-existing nAb titers were of low magnitude and differences in pre-existing anti-vector immunity did not impact on vaccine immunogenicity.

Priming with the first ChAd3 vaccination resulted in an expansion of nAb in all but two individuals (Fig. 4a). However, nAb titers (that were higher at baseline in arm A3) were boosted to significantly higher levels in arm A3 than arms A2/A4, and, importantly, these titers remained significantly higher at the time of reboosting with a second ChAd3-NSmut vaccination (short interval gp A3 GM 1,037 vs. long interval gp A4 GM 137; *p* = 0.03. Fig. 4a). Overall, the fold increase in HCV-specific T cell responses before and after 2nd ChAd3 administration negatively correlated with ChAd3 nAb titers at the time of vaccination (Fig. 4c), which suggests that high nAb titers at the time of reboosting with ChAd3-NSmut could have resulted in the observed poor/non- response to the encoded antigen after a 2nd ChAd3 vaccination in group A3 (Fig. 2a).

In assessing the hexon-specific T cell response, no correlation with vaccine immunogenicity was seen. Baseline hexon T cell responses were not significantly different between Arms A3 and A4 (GM 155 vs. 89, *p* = 0.24; Fig. 4d) and there was no correlation between the magnitude of the hexon-specific T cell response at the time of ChAd3 administration and the induction (Fig. 4e) or expansion (Fig. 4f) of HCV-specific T cell response by vaccination in any groups. Taken together these data suggest that the humoral, rather than the T cell component, of the anti-adenovirus response may interfere with re-administration of Ad vectors when anti-Ad antibodies reach high levels after Ad priming vaccination.

### Reboosting changes the phenotype of the vaccine-induced memory T cell populations

**Fig. 5:**
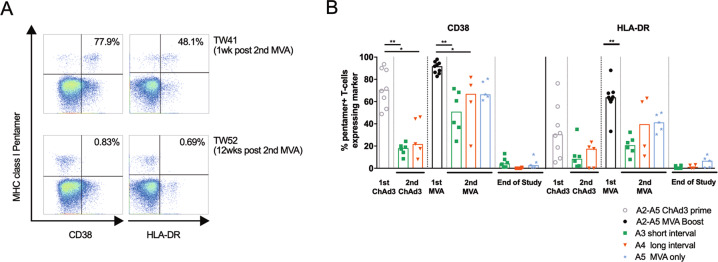
T cell activation is strongest after MVA vaccination but it is not affected by the length of the interval since last NS encoding vaccination. **a** Example plots (CD8^+^ T cells) showing pentamer vs. activation marker (arm A5) 1 week and 12 weeks post second MVA-NSmut vaccination (see Supplementary Fig. 4 for full gating strategy). Percentage of pentamer^+^ cells expressing the activation marker is shown. **b** Ex vivo MHC class I pentamer staining was performed on all HLA-A*01^+^ or A*02^+^ individuals (NS3_1435–1443_, NS3_1406–1415,_ NS3_−1073–1081,_ see methods) from arms A2-A5. PBMC were co-stained with individual pentamers and antibodies against the CD8^+^ T cell surface activation markers CD38 and HLA-DR. The percentage of pentamer^+^ cells expressing these markers is shown, bars at median. **b** Kruskal–Wallis one-way ANOVA with Dunn’s correction comparing all group means for 1st and 2nd ChAd3-NSmut vaccination, and for 1st and second MVA vaccination for CD38 and for HLA-DR, significant differences shown. *P < 0.05; **P < 0.005.

We have previously shown that vaccine-induced CD8^+^ T cells were highly activated (as measured by CD38 and HLA-DR expression on MHC class I pentamer^+^ T cells) immediately after ChAd3-NSmut prime and MVA-NSmut boost vaccinations. The strongest expression of activation markers on HCV-specific T cells coincides with the peak in magnitude of the response 1 week after MVA-NSmut vaccination^11^. We therefore investigated whether T cell activation status correlates with the expansion of HCV-specific T cells response on reboosting. Only a small subset of HCV-specific T cells expressed activation markers after a second round of ChAd3-NSmut, irrespective of the time interval since the initial prime-boost vaccinations (Fig. 5). However, reboosting with a second MVA-NSmut vaccination, given alone or after a second ChAd3-NSmut, resulted in expression of activation markers on the majority of HCV-specific T cells (arm A4 or A5 vs. arm A3). Activation markers expression was rapidly lost with contraction of the HCV-specific T cell response, with little expression of these markers being seen in the resting memory populations at the end of the study. A higher expression of activation markers by HCV-specific T cells was seen in all cases where an expansion of the HCV-specific T cell response occurred.

**Fig. 6:**
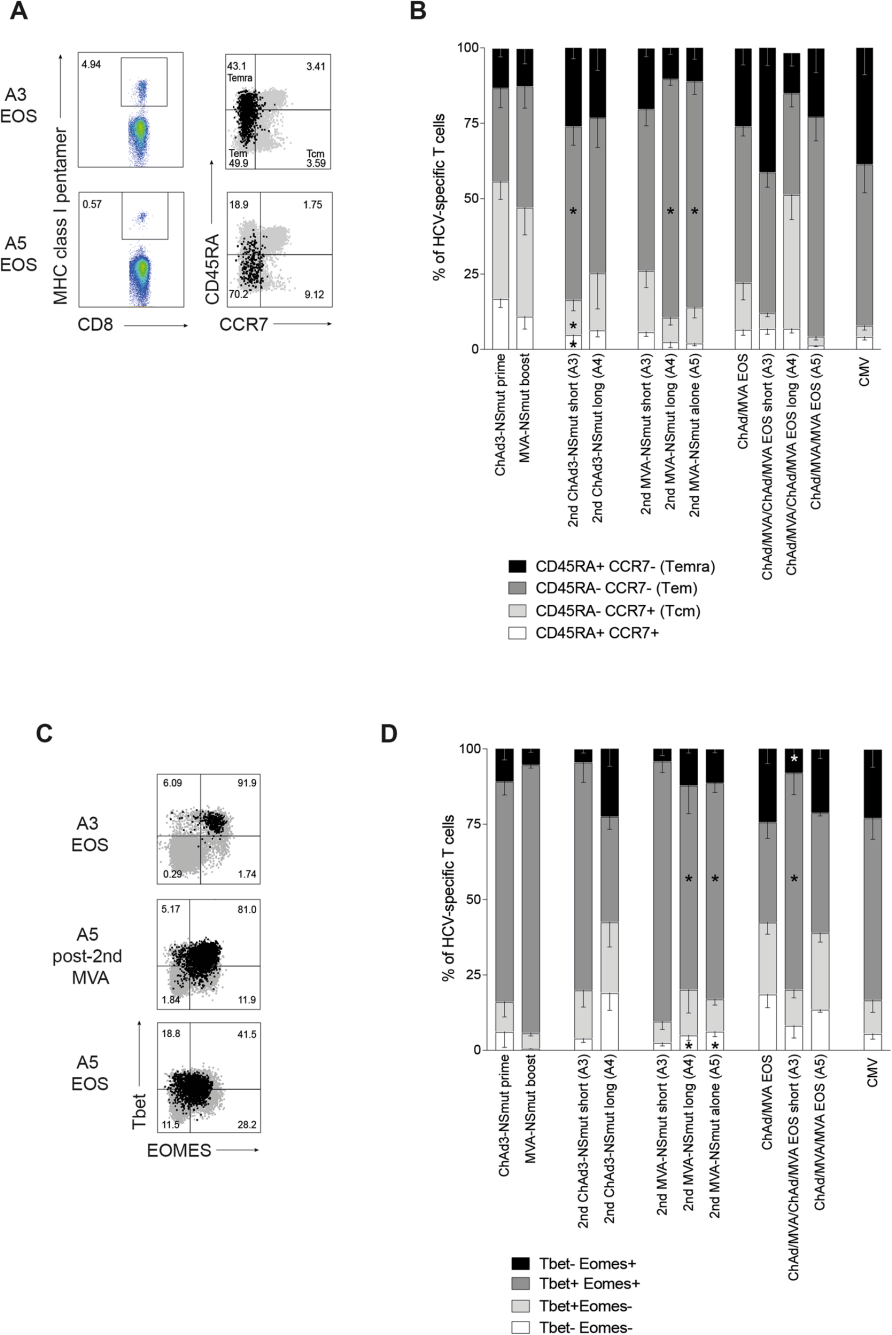
Reboosting alters the proportions of T cell memory subsets. (**a**–**d**) PBMC from HLA-A*01^+^ or A*02^+^ individuals were co-stained ex vivo with individual MHC class I pentamers and antibodies against surface markers CD45RA, CCR7 (**a**, **b**), or intranuclear transcription factors Tbet and Eomes (**c**, **d**). **a** Example FACS plots (pentamer gating and overlay of total CD8^+^ in grey and pentamer^+^ in black; see Supplementary Fig. 4 for full gating strategy). **b** Stacked bars showing the proportion of pentamer^+^ T cells with a given phenotype at the peak of the T cell response (1–4 weeks post vaccination, according to IFNγ ELISpot) after each vaccination and at the end of the study (EOS; *n* = 4–13 per pie). Where CMV responses were detected by IFNγ ELISpot in HLA-A*02^+^ individuals an immunodominant CMV pentamer was used in parallel (*n* = 10). **c** Example FACS plots (overlay of total CD8^+^ in grey and pentamer^+^ in black). **d** Stacked bars showing the proportion of pentamer^+^ T cells co-expressing Tbet and Eomes at the peak of the response (1–4 weeks post vaccination, according to IFNγ ELISpot) after each vaccination and at the end of the study (*n* = 4–13 per pie). The proportion of CMV-specific T cells showing each subset phenotype is also shown (*n* = 6). **b**, **d** Mean, bars at standard error of mean. Kruskal–Wallis unpaired non-parametric Anova comparing ChAd3-NSmut prime vs. 2nd ChAd-NSmut short (A3) or long (A4), MVA-NSmut boost vs. 2nd MVA-NSmut short (A3) or long (A4) or alone (A5), ChAd3-NSmut/MVA-NSmut EOS vs. ChAd/MVA/CMVA EOS short (A3) or ChAd/MVA/MVA (A5). *P < 0.05.

The co-expression pattern of the lymph node homing marker CCR7 and CD45 protein tyrosine phosphatase isoform CD45RA can be used to identify different memory subsets of T cells^30^. We co-stained HCV-specific T cells with these markers to investigate whether re-vaccination alters the proportions of T cell memory subsets across the trial. At the peak of the T cell response to initial ChAd3-NSmut prime and MVA-NSmut boost vaccinations HCV-specific T cells were a mixed population of effector memory (Tem; CD45RA^−^ CCR7^−^) and central memory (Tcm; CD45RA^−^ CCR7^+^) subsets (Fig. 6). When re-vaccinating with ChAd3-NSmut after a short (**A3**) or long (**A4**) interval most HCV-specific T cells were Tem and Temra (terminally differentiated effector memory, CD45RA^+^CCR7^−^), with few Tcm. The intensive vaccination regimen of ChAd3-NSmut/MVA-NSmut/ChAd3-NSmut/MVA-NSmut all 8 weeks apart lead to a larger proportion of Temra and Tem T cells with a concomitant reduction in Tcm and naive-like memory T cells when compared to other regimens (Fig. 6a, b). Re-vaccination with a second MVA-NSmut also tended to produce more Tem, and fewer Tcm and Naïve-like T cells, similar to the proportions seen in memory responses to CMV (Fig. 6b)^31^. Overall, where a stronger T cell expansion was seen, as in Arm A4 and A5, a larger Tem population was seen immediately after vaccination, and reboosting lead to a more terminally differentiated population of T cells.

Co-expression patterns of the T-box transcription factors (TF), T-bet and Eomesodermin (Eomes) coordinate both T cell memory formation and effector function, in particular IFNγ expression and cytolytic potential^32–35^. We assessed their expression on vaccine-induced HCV-specific T cells to see if re-vaccination drives a TF profile indicative of terminal differentiation or exhaustion. After all vaccinations, HCV-specific T cells were dominated by Tbet^+^ (T-box transcription factor TBX21) Eomes^+^ (Eomesodermin) T cells, indicative of Tem and Temra^31^ subsets, with few Eomes^−^Tbet^−^ (Tcm and Naïve-like), in line with surface marker expression (Fig. 6c, d). Vaccine-induced T cells showed similar TF co-expression to functional memory T cells seen after CMV infection (Fig. 6c, d). There were few differences in Tbet and Eomes expression between memory T cells elicited by different re-vaccination schedules. Although still the dominant population, after a second MVA vaccination fewer T cells were Tbet^+^Eomes^+^. It is important to note that even after reboosting T cells were not driven to downregulate Tbet whilst retaining Eomes, which is a TF profile indicative of T cell exhaustion by chronic viral infection (Fig. 6c, d)^35^.

Overall, ChAd-NSmut/MVA-NSmut induced T cells are predominantly effector memory similar to highly functional memory response to CMV. The overall balance of T cell memory subsets is only subtly altered by repeated vaccination, and functionality and TF profile were not adversely affected by reboosting vaccination.

### A ten-fold lower dose of MVA-NSmut can be used without impacting the magnitude, breadth, functionality, or phenotype of the vaccine-induced T cell response

The possibility of inducing a comparable transgene-specific immune response whilst using a lower dose of MVA than is currently being used (1–5 × 10^8^ pfu;^11,36–42^ would circumvent some of the difficulties and costs in manufacturing MVA and may reduce vaccine reactogenicity.

A dose de-escalation study was performed to assess the impact of lowering the MVA dose ten- or a hundred-fold on reactogenicity and immunogenicity. Healthy volunteers received a full priming dose of ChAd3-NSmut (2.5 × 10^10^ vp) and then 8 weeks later volunteers received a medium dose of 2 × 10^7^ pfu MVA-NSmut (**arm A6**) or a low dose of 2 × 10^6^ pfu MVA-NSmut (**arm A7**). Following vaccination with a medium or low dose of MVA-NSmut very few AEs were observed (Supplementary Fig. 2). Only 11 and 7 AE’s were experienced in the medium (all Grade 1) and low dose MVA arms respectively. A trend for a reduced number of AEs with lower doses of MVA-NSmut boosting vector was seen (Supplementary Table 4**)**.

**Fig. 7:**
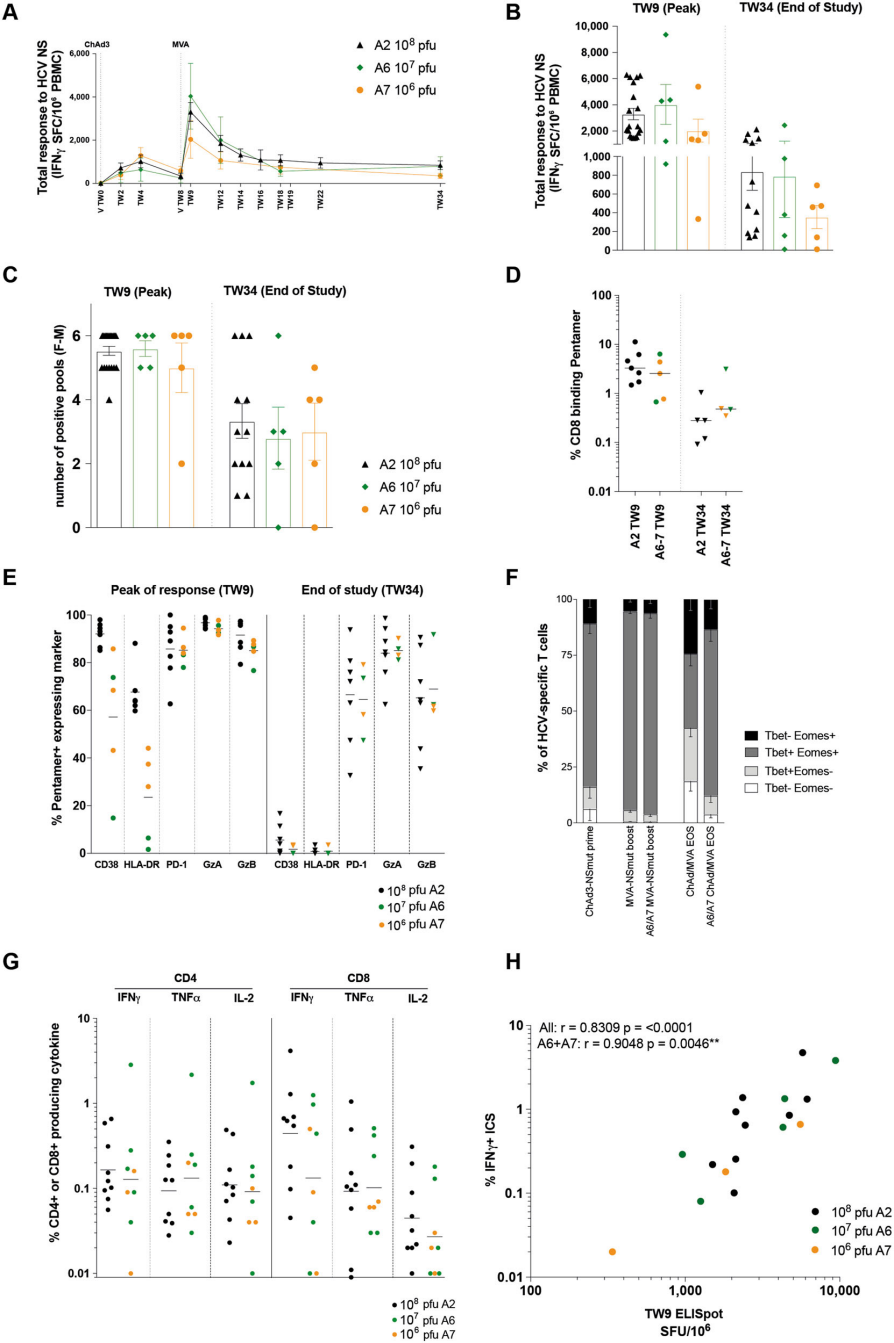
The magnitude, breadth, functionality, and phenotype of vaccine-induced HCV-specific T cells are unchanged when using a medium MVA-NSmut dose when compared to high dose. **a**–**h** Healthy volunteers receiving ChAd3-NSmut prime vaccination were boosted with high (10^8^ pfu; black dots, arm A6), medium (10^7^ pfu; green dots, arm A7), or low dose (10^6^ pfu; orange dots) MVA-NSmut vaccination 8 weeks later (Supplementary Table 1). **a**, **b:** the summed *ex vivo* IFNγ ELISpot response to HCV NS encoded in the vaccine. **a** Kinetics of the HCV-specific T cell response across the vaccine trial (group mean). **b**–**e** Comparison of peak (1-week post-MVA-NSmut, TW9) and memory (end of study [EOS], TW34) (**b**) HCV-specific T cell response, (**c**) breadth of the HCV-specific T cell response (number of positive pools, see methods), (**d**) percentage of CD8^+^ T cells binding MHC class I pentamers ex vivo (NS3_1435–1443_, NS3_1406–1415_), and (**e**) percentage of HCV-specific pentamer^+^ T cells expressing CD38, HLA-DR, PD-1, granzyme A (GzA) or granzyme B (GzB). **f** The percentage of pentamer^+^ T cells co-expressing Tbet and Eomes at the peak of the T cell response after ChAd3-NSmut prime (TW2-4), after MVA-NSmut (TW9) and at EOS (arms A6 and A7 combined; TW34). **g** The percentage of CD4^+^ or CD8^+^ T cells producing IFNγ, TNFα or IL2 at the peak of the T cell response (TW9). **h** Correlation between the magnitude of HCV-specific T cell response induced by vaccination as measured by response to peptide pool G by ELISpot and percentage pentamer^+^ (immunodominant epitope in pool G, HLA-A*02-restricted NS3_1406–1415_). Spearman r calculated for all data combined or for A6 and A7 data combined. **a**–**c** mean ± standard error of mean. **d**, **e**, **g** Bars at median. **b**, **c**, **e**, **g** Kruskal–Wallis one-way Anova with Dunn’s correction for multiple comparisons, all non-significant. **d** Mann–Whitney *t* test non-significant.

Importantly, no significant reduction in the HCV-specific T cell response to MVA-NSmut boost was observed at any time point during the trial when medium or low dose MVA-NSmut vaccinations are used (Fig. 7), although there is a trend for a lower peak and memory HCV-specific T cell response with low dose MVA-NSmut (Fig. 7a, b). No reduction in the breadth of the vaccine-induced T cell response (as determined by the number of positive pools in the IFNγ ELISpot) at its peak or for the memory T cell response was seen when lowering the dose of MVA-NSmut (Fig. 7c). The magnitude of the T cell response to individual immunodominant HLA-A*01 and HLA-A*02 restricted epitopes was also comparable in volunteers who received a medium or low dose of MVA-NSmut, both at the peak of the response and at the end of the trial (Fig. 7d). T cells targeting these epitopes appeared to have a reduced expression of activation markers just after vaccination when given medium dose or low dose MVA-NSmut but showed comparable expression of cytolytic markers and PD-1 (Fig. 7e). HCV-specific T cells induced by lower doses of MVA also showed similar TF co-expression to high dose MVA vaccination (Fig. 7f). The cytokine production of CD4^+^ and CD8^+^ HCV-specific T cells on peptide stimulation at the peak of the T cell response (TW9) was also comparable in those that received medium or low dose MVA-NSmut (Fig. 7g, h).

**Fig. 8:**
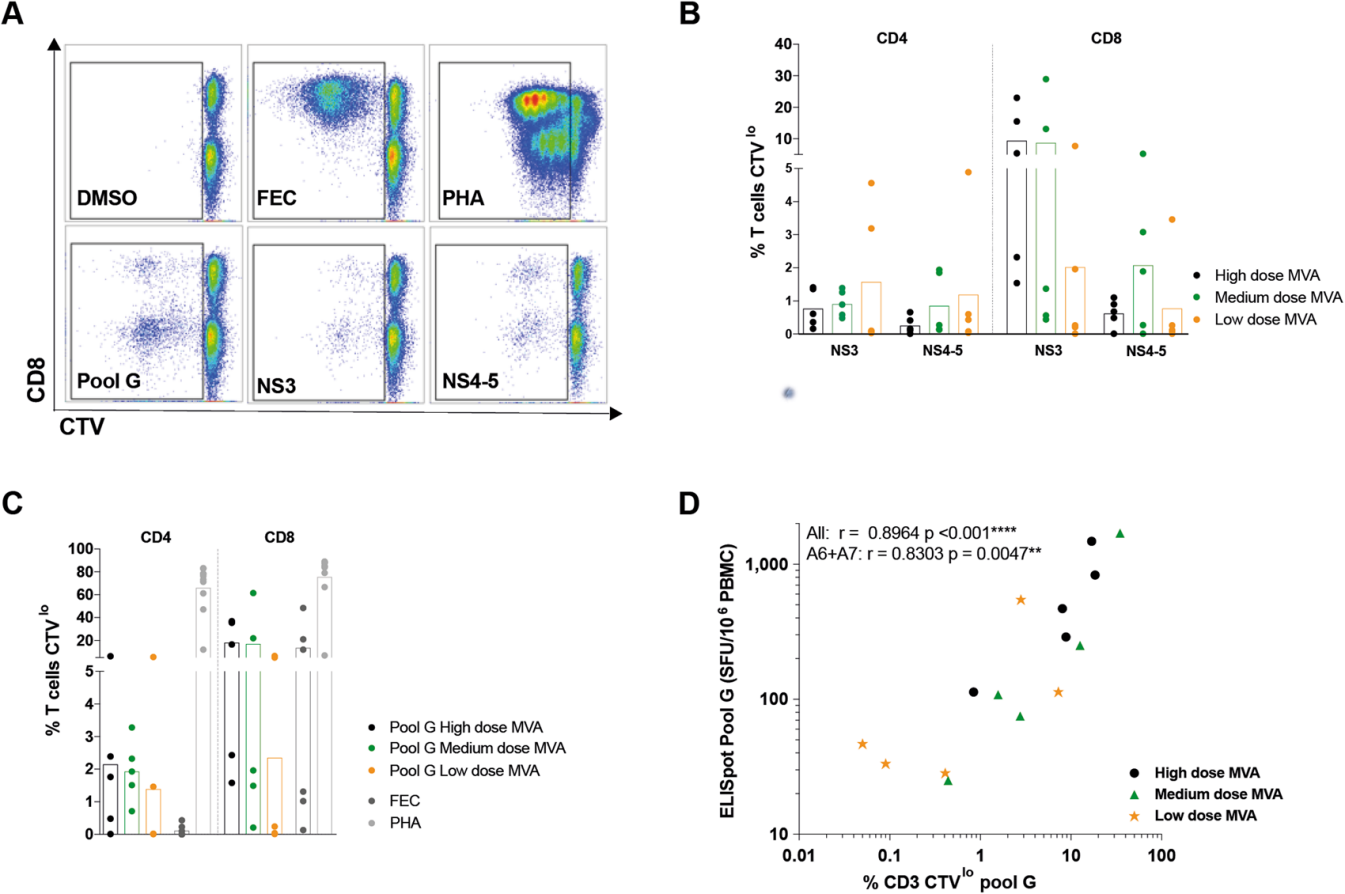
Full and medium MVA-NSmut dose induced HCV-specific T cells with equivalent proliferative capacity. **a** Example plots of CTV dilution against CD8^+^ (gated on lymphocytes/singlets/live/CD3^+^) for each stimulation (see Supplementary Fig. 4 for full gating strategy). **b**–**d** The percentage of CD4^+^ or CD8^+^ T cells that proliferated (CTV^lo^) over 5 days in response to stimulation with (**b**) peptides covering the NS region of HCV (NS3 or NS4-5), (**c**) with a single immunodominant pool covering part of NS3h (pool G), with immunodominant MHC-class I restricted peptides from Flu, EBV, and CMV (FEC) or PHA (phytohemagglutinin). PBMC isolated at the end of the study were stimulated (11–32 weeks post-MVA-NSmut boost vaccination). **d** The percentage of CD3^+^ T cells proliferating over 5 days in response to pool G was correlated with the T cell response to pool G by ELISpot at the same time point (end of study). **b**, **c** Kruskal–Wallis one-way Anova with Dunn’s correction between high, medium and low dose MVA groups for NS3, NS4-5 and pool G for CD4^+^ and CD8^+^ T cells, all non-significant. **d** Spearman r calculated for all data combined or for A6 and A7 data combined.

Finally, we assessed the ability of T cells induced by medium and low dose MVA vaccination to expand on further antigen encounter. CD4^+^ and CD8^+^ T cells induced by a medium dose of MVA had a robust in vitro proliferative response to HCV peptides that was comparable to T cells induced by high dose MVA (Fig. 8). The proliferative capacity of the T cells induced by all doses of MVA after stimulation with peptides covering the immunodominant pool (G, covering NS3 helicase) correlated well with the magnitude of the HCV-specific T cells response by ELISpot (Fig. 8); this suggests that despite the slightly lower magnitude of response (seen by ICS and ELISpot) there was no intrinsic proliferative defect in T cells induced by low dose MVA, as T cell proliferation was directly proportional to the magnitude of the response.

Overall, a hundred-fold reduction in MVA dose may impact the magnitude of the HCV-specific T cells response, however, a ten-fold reduction in dose did not alter the magnitude, breadth, functionality, or phenotype of the vaccine-induced T cell response.

## Discussion

Viral vectored vaccines have become increasingly safe and efficacious as our understanding of the innate and adaptive immune responses to both the vector and the inserted transgene have developed, however, many questions remain as to how best to employ these potent T cell inducers. To produce affordable, clinically practical, yet highly efficacious regimens optimisation of dose, timing, and vector combination is required.

Here we first investigated the possibility of enhancing the encoded transgene-specific memory T cell response by reboosting volunteers that had previously received a prime-boost vaccination with ChAd3-NSmut/MVA-NSmut with further rounds of ChAd3-NSmut and MVA-NSmut. As with single administrations, re-vaccination with ChAd3 or MVA was safe and well tolerated. The type, number, and severity of AEs were similar for first or second vaccinations with both ChAd3-NSmut or MVA-NSmut, and as with initial vaccination MVA was more reactogenic than ChAd3. Importantly, ChAd3 and MVA are no more reactogenic when re-administered to the same healthy volunteers.

The activation status and the re-expansion of HCV-specific T cells was greatly limited when the second round of ChAd3-NSmut/MVA-NSmut was given shortly after the initial prime boost (all vaccines 8 weeks apart). However, if reboosting is delayed strong recall responses are seen. When the second round of ChAd-NSmut/MVA-NSmut was given a year after the initial prime-boost vaccinations the HCV-specific T cell response expanded to a similar magnitude as that seen at the peak after the initial vaccinations. We also showed that a simplified reboosting regimen employing a second MVA-NSmut alone appears to be as immunogenic as reboosting with ChAd3-NSmut/MVA-NSmut.

We assessed whether the re-expansion of T cells targeting the HCV NS transgene, which was improved with a long interval between rounds of ChAd-NSmut, could be explained by the differential induction and maintenance of nAbs to ChAd3, or through the HCV-specific T cell differentiation state at the time of administering the second ChAd3-NSmut vaccination. Anti-Ad vector nAbs have been shown to limit transgene expression and T cell induction after vaccination in mice and humans^29,43–46^ and extending the period between ChAd3-NSmut vaccinations may allow nAbs to wane and reduce anti-vector immunity. We found that pre-existing nAb titers to ChAd3 were low in all volunteers as expected, with no correlation between pre-existing nAb titers and vaccine immunogenicity. However, following the first ChAd vaccination, very high levels of ChAd3 nAbs were generated. Due to the small sample size, and the fact that by chance nAbs were higher at baseline in the short interval group (arm A3), it was not possible for us to directly conclude that a short interval between ChAd vaccinations directly resulted in higher nAbs at the time of the second ChAd vaccination. However, at these high levels of anti-Ad nAbs, there was a clear negative correlation between nAb Ad titer and the subsequent re-expansion of HCV-specific T cells following the second ChAd3 vaccine. Therefore, future studies may evaluate nAbs as a potential predictor of vaccine responsiveness in reboosting strategies. It is well recognized that T cell response to adenovirus predominantly target the hexon protein and are cross-reactive amongst serotypes, as demonstrated by the vigorous expansion of anti-vector T cell responses upon vaccination detected by Ad5 Hexon stimulation here. However, there may be minor T cell responses to other viral proteins or ChAd3 hexon-specific T cell responses escaping our assessment. These limitations result in an imperfect ability to strictly determine the role and main players of anti-vector immunity in impacting the ability to respond to ChAd3 vector re-administration in our study.

The description of T cell memory subsets and the division of labor within a T cell response has led to the understanding that certain subsets are the main mediators of protection after vaccination and during the course of natural infection^47–50^. A longer interval between vaccinations may allow transgene-specific T cells to fully differentiate into memory T cells that can optimally respond to antigen re-exposure. We have previously observed that the HCV-specific T cells response contracts for at least 14 weeks after MVA-NSmut boost in most healthy volunteers and T cells continue to change phenotype and differentiate up to at least 26 weeks post-MVA-NSmut^11^. In volunteers who received ChAd3-NSmut/MVA-NSmut/ChAd3-NSmut/MVA-NSmut, all 8 weeks apart, T cells assayed long term after the last vaccination showed a more terminally differentiated phenotype, whereas a larger population of Tcm persisted when reboosting was delayed. A large proportion of the HCV-specific T cells induced by all Ad and MVA containing regimens were Tem phenotype, a subset that is usually induced when T cells see persistent low-level exposure to antigen^31,51^. However, CMV-derived vectors also induce predominantly Tem CD8^+^ T cells and promising efficacy studies in non-human primates of rhesus-derived CMV vectors encoding HIV-1 antigens have prompted a paradigm shift to a preference for vaccine induction of circulating or tissue-resident memory T cells with immediate effector function^52–55^. Reboosting after a long interval for CD4^+^ and CD8^+^ T cells, and after a short interval for CD8^+^ T cells, also led to a larger proportion of polyfunctional transgene-specific T cells than after the initial ChAd3-NSmut/MVA-NSmut prime/boost vaccinations. The T cell subset that will offer best protection against HCV infection is not yet known, however, this work highlights the exciting possibility of tailoring the timing and combinations of vectors within a regimen to elicit a specific mix of T cell memory subsets.

We measured the expression of two transcription factors that coordinate many genes involved in differentiation and function of CD8^+^ T cells (Tbet and Eomes^56–58^). Tbet^+^Eomes^−^ CD8^+^ T cells have been described as a progenitor population with an intrinsically low homeostatic turnover that give rise to Tbet^+^Eomes^+^ cells when activated^35^. Repeated exposure to antigen above a certain threshold appears to strain the regenerative capacity of Tbet^+^Eomes^−^ T cells, and drives the accumulation of exhausted Tbet^−^Eomes^+^ T cells; this has been observed in the setting of chronic HBV, HCV, and HIV-1^35,59,60^. It is important to note here that all vaccine regimens induced functional effector and memory T cell responses, similar in phenotype to highly functional CMV-specific T cell memory^31^, which retained their Tbet expression, and there was no sign of exhaustion in functional assays or by TF expression.

Due to difficulties in manufacturing MVA vectors, a lower dose of MVA may be required for large-scale vaccine programs. A lower dose may also have the added benefit of reducing the reactogenicity of the vector and of lowering vaccine costs^23^. Here we perform the first in depth characterization of the transgene-specific T cell response induced by a ten-fold (10^7^ pfu) and a 100-fold (10^6^ pfu) lower dose of MVA than that currently used in most phase I trials. We found that 10^7^ pfu MVA induces an equivalent T cell response to 10^8^ pfu MVA in terms of T cell magnitude, breadth, kinetics, T cell phenotype (surface and transcription factor expression) and functionality (proliferative capacity, cytokine production, and cytolytic potential). Although not statistically significant, the magnitude of the HCV-specific T cell response may be reduced at its peak and at the end of the study when a 100-fold lower dose (10^6^ pfu) is used. In addition, the number of AEs per vaccination was reduced for each severity, and percentage of individuals showing a given AE was lower for many AEs (e.g. moderate/severe pain, swelling, Erythema, Fever, Myalgia Fatigue etc.) when 10^7^ or 10^6^ pfu MVA was administered. This first-in-man dose titration of MVA heterologous boost, shows that a ten-fold reduction, compared to current standard dosing is equally immunogenic and better tolerated; a larger trial evaluating this dose of MVA is now required.

Several global health priorities (infectious agents and cancers) have shown an inherent ability to evade antibody vaccines and there is renewed optimism for the potential of T cell-based vaccines^55,61^. Ad and MVA based vaccines are in development for most, if not all, of these health priorities and optimisation of T cell vaccine regimens is essential to aid in their development. In conclusion, we show that transgene-specific T cells responses can be boosted by further ChAd and MVA vaccinations and that the magnitude of the response is most effectively boosted when a long interval of at least 30 weeks is given between the initial vaccine round and reboosting. We also show that the phenotype of the vaccine-induced T cells can be altered by repeat vaccinations. However, no signs of T cell exhaustion are seen, even with the most intensive vaccine regimen. We also show importantly that a 10-fold lower dose than is currently in testing for MVA vaccine vectors is as immunogenic and should be used in future trials.

## Methods

### Ethics and regulatory approval

This clinical trial is registered in the ClinicalTrial.gov database (ID: NCT01296451, December 2010) and arms A1 and A2 of this clinical trial have been published previously in^11^. Approvals for the study were granted by the UK National Research Ethics Service, (NRES Committee South Central—Oxford REC A, REC reference number 10/H0604/45) and the UK Medicines and Healthcare Products Regulatory Agency (EudraCT no. 2009-018260-10). GCP compliance was independently monitored by the University of Oxford Clinical Trials and Research Governance office. A multinational independent data safety monitoring committee (DSMC) provided safety oversight.

### Volunteer demographics and study arms

Four volunteers in arm A2 were enrolled in to Arm A4 and received a second round of ChAd3-NSmut and MVA-NSmut vaccination 47–92 weeks after first receiving ChAd-NSmut prime vaccination (Fig. 1**;** Supplementary Table 1). The two primary endpoints of this study are vaccine safety and immunogenicity. All volunteers gave written informed consent before enrolment, and the studies were conducted according to the principles of the Declaration of Helsinki and in accordance with Good Clinical Practice. Volunteers were recruited at the CCVTM (Centre for Clinical Vaccinology and Tropical Medicine), Churchill Hospital, Oxford. Study arms and vaccination regimes are detailed (Supplementary Table 1). For this first-in-man Phase I study no randomisation or blinding of participants and data was used. A dose escalation for ChAd3-NSmut is described in^62^. The high MVA dose was selected on the basis of previous use of MVA vectors in human studies^11,63,64^. Vaccines were administered intramuscularly in the deltoid region of the arm.

Volunteers were observed for 1 to 3 hours after vaccination and then they systematically documented all symptoms and recorded a daily oral temperature. Solicited and un-solicited local and systemic AEs were collected in diary cards and recorded in case report forms. Both exposure to HCV (defined as HCV antibody and HCV RNA positivity) and recent intravenous drug use were study exclusion criteria. HCV antibodies, quantitative HCV RNA, and T cell response to HCV antigens were all undetectable at baseline. There were no signs or symptoms of viral hepatitis or an increase in liver enzymes during the study. One volunteer withdrew from follow up in arm A5 at trial week 14, prior to receiving reboosting vaccination with MVA-NSmut (volunteer 341). Safety data and immunogenicity data are included for this volunteer up to trial week 14.

### Vaccine constructs

Non-replicative chimpanzee-derived adenoviral vaccine vectors (ChAd3) encoding the NS3-5B (NS) region of BK strain HCV (1985 amino-acids; accession number M58335; genotype 1b) has been described previously^11,28,65^. Generation of a MVA construct containing the same NS region transgene has been previously described^11^. The NS region contains a point mutation (GlyAspAsp to AlaAlaGly) at positions 1711 to 1713 in the catalytic site of the NS5b which inactivates this RNA polymerase without reducing the expression of HCV proteins or processing of the polypeptide by the NS3 encoded protease and is therefore referred to as NSmut^66^.

### Peptides and antigens

494 peptides 15 amino-acids long overlapping by 11 amino-acids (BEI Resources) were divided into 6 pools (F-M) corresponding to NS3p, NS3h, NS4, NS5A, NS5B I, NS5B II (mean 82, range 73–112 peptides/pool) matching HCV genotype 1B strain BK. Peptides covering the complete sequence of the Hexon protein of Ad5 (15 amino-acids long overlapping by 11 amino-acids; UniProt ID: P04133; Miltenyi) were used to assess anti-vector immunity. Ad5 hexon and ChAd3 hexon sequence identity 87% and homology 91%; 95% identity and 97% homology when excluding the hypervariable regions. Peptides were used at a final concentration of 3 µg/ml and 1 µg/ml per peptide for ELISpot and Intracellular cytokine staining, respectively.

### IFNγ-ELISpot assays

Unless otherwise stated culture medium for human T cells was R10: RPMI medium (GibcoBRL) supplemented with 10% by volume heat inactivated (1 h 64 °C) fetal calf serum (FCS; Hyclone, 1% by volume 200 mM L-glutamine (GibcoBRL) and 1% by volume 100x penicillin and streptomycin solution (GibcoBRL).

IFNγ-ELISpot assays were performed ex vivo in triplicate with 2 × 10^5^ peripheral blood mononuclear cells (PBMC)/well in R10 as previously described^62^. PBMC were separated via density gradient (Lymphoprep) and counted using a Guava Personal Cell Analyser (Merck Millipore). Millipore AIP plates (MSIP54510) were coated at 4 °C with anti-human IFNγ monoclonal antibody (5 μg/ml in PBS; MAbTech clone 1-D1K) overnight. Plates were incubated for 3 h at room temperature with biotinylated mouse anti-human IFNγ monoclonal antibody (0.5 mg/ml; MAbTech clone 7-B6-1) diluted in assay diluent (0.5% BSA/PBS). The plate was then incubated with alkaline phosphatase-conjugated anti-biotin antibody diluted 1:750 in assay diluent (Vector Laboratories) for 2 h and finally incubated at room temperature with sterile filtered BCIP/NBT substrate solution (Pierce cat. 34042). Plates are air-dried overnight and read on an AID ELISpot plate reader. Internal controls were R10 alone (without cells) and DMSO (negative controls) and Concavalin A, FEC (HLA class I-restricted peptides from influenza, Epstein-Barr virus, and CMV), and CMV lysate (Virusys Corporation USA; positive controls).

A robust positive cut off for HCV-specific T cell responses in the IFNγ-ELISpot assay was defined in^62^. For a positive response, the mean of antigen wells was determined (i) to be greater than 48 SFCs/10^6^ PBMCs and (ii) to exceed 3× background. Background wells (medium only, cells + DMSO) were typically zero to four spots. Total NS response was calculated by summing responses to all positive pools (NS3p-NS5B II) after correcting for background.

### Intracellular cytokine staining

PBMC at 1 × 10^6^ cells in 100 µl R10 were stimulated with peptide pools (F+G+H = NS3/4, I+L+M = NS5A/B) or PMA (phorbol 12-myristate 13-acetate)/ionomycin (50 and 500 ng/ml respectively), or unstimulated (DMSO). Brefeldin-A was added (10 µg/ml) 1 h later, cells were incubated overnight (37 °C), stained with fixable-NIR live/dead dye (Life Technologies), fixed (1% paraformaldehyde) and permeabilised (Foxp3 Fix/Perm kit, BD Biosciences) then stained with the following antibodies at room temperature for 30 mins: CD3-PO (Pacific Orange; Invitrogen, UCHT1), CD4-Qdot 605 (Invitrogen, S3.5) CD8-PB (Pacific Blue; BD biosciences, RPA-T8), IFNγ-Alexa Fluor 700 (BD biosciences, XMG1.2), IL-2-APC (BD biosciences, 5344.111), and TNFα-PE-Cy7 (BD biosciences, MAb11). Flow cytometry was performed using a BD LSRII and analysis by FlowJo (Tree Star) version 10.4.1. All ICS data are corrected for background (cytokine production in paired DMSO wells).

Pestle version 1.8 and Spice version 6.0 were used for background subtraction, data formatting, and data visualization for polyfunctionality assessment of ICS data. Samples with a total T cell response to HCV NS of <0.025% of CD4^+^ or CD8^+^ T cells were excluded. Pie base, means.

### FACS based proliferation assay—Cell Trace Violet dilution

To assess the proliferation of T cells after 5-day stimulation with peptides or PHA, frozen PBMC were thawed and washed in PBS and 1–10 × 10^6^ PBMC were stained with 2.5 µM Cell Trace Violet (ThermoFisher) for 10 mins in the dark at room temperature. 10x volume of ice cold RH10 (media containing 10% human ab serum) was added and cells were kept on ice for 5 mins. PBMC were pelleted and resuspended in 10mls of RH10, transferred to a new tube and incubated at 37° C for 5 mins. PBMC were then washed again in PBS and plated 1 × 10^6^ cells in 0.5 ml RH10 per well in a 48 well plate with the relevant stimulation; peptides covering HCV non-structural region were added at 1 µg/ml per peptide; immunodominant MHC-class I restricted peptides from Flu EBV and CMV (FEC) were added at 1 µg/ml per peptide; PHA (phytohemagglutinin) was added at 2 µg/ml. After 5 days stimulation PBMC were harvested from the plate, washed in PBS, and were surface stained as above with fixable Live/dead and antibodies against CD3-FITC (Biolegend, UCHT1), CD4-Alexa fluor-700 (BD biosciences, RPA-T4) and CD8-PE-Cy7 (BD biosciences, RPA-T8) and were analyzed by flow cytometry as above.

### HLA Class I pentamer staining

Frozen PBMC were thawed and stained ex vivo with HLA*0201 or HLA*0101 PE-labelled pentamers (Proimmune Oxford; 20 min in PBS, RT), stained with LIVE/DEAD Fixable Near-IR Dead Cell Stain Kit (Thermo Fisher; 20 min RT), fixed (1% formaldehyde in PBS, 20 mins RT) then stained with: CD3-PO (Pacific Orange; Invitrogen, UCHT1), CD8–PB (Pacific Blue; BD biosciences, RPA-T8), and either stained with CCR7-PeCy7 (CD197; BD biosciences, 3D12), CD45RA-FITC (BD biosciences, HI100), CD38-PerCP-Cy5.5 (Biolegend, HIT2), HLA-DR-AlexaFlour700 (BD biosciences, G46-6) for 30 min RT; or permeabilised (10x permeabilisation buffer; ebiosciences) and stained with PD1-PeCy7 (BD biosciences, EH12.1), Perforin-FITC (BE biosciences, dG9), Granzyme B- Alexa fluor700 (BD biosciences, GB11) and Granzyme A-PerCpCy5.5 (Biolegend, CB9) 30 min RT. For intranuclear staining, PBMC were stained as above for pentamers and Live/Dead dye, and then surface stained with: CD3-Pacific-orange, CD8-PB (30 min RT). PBMC were then fixed (1 h RT) and permeabilised using the Foxp3/Transcription Factor Staining Buffer Set (ebiosciences; 45 min RT) and stained with eomes-eFluor660 (eBiosciences, WD1928) and Tbet-BV605 (Biolegend, 4B10).

### nAbs to ChAd3 vector

An assay measuring the neutralising antibodies against ChAd3 vector was performed as previously described using recombinant ChAd3 expressing the reporter geneSEAP^16,67^.

### Statistical analysis

Data were assumed to have a non-Gaussian distribution. Non-parametric tests were used throughout; paired tests within an individual (Wilcoxon matched pairs signed rank test) or unpaired tests between individuals (Mann-Whitney). For unpaired multiple comparisons Kruskal–Wallis one-way Anova with Dunn’s correction was used. For correlations, Spearman’s r test was used. A *p* value <0.05 was considered significant. Prism v. 7.0e (Graphpad) for Mac was used for analysis. * = *p* ≤ *0.05;* ** = *p* ≤ *0.01*; *** = *p* ≤ *0.001*; **** = *p* < *0.0001*.

### Reporting summary

Further information on research design is available in the Nature Research Reporting Summary linked to this article.

## Supplementary information

Supplementary Information

Reporting Summary

## Data Availability

The data that support the findings of this study are available from the corresponding author L.S. upon reasonable request. This study did not generate any unique code or datasets other than those included in this published article (and its Supplementary Information files).
