# Supplementary material for: Optimising T cell (re)boosting strategies for adenoviral and modified vaccinia Ankara vaccine regimens in humans
Source: NPJ Vaccines. 2020 Oct 12;5:94. doi: 10.1038/s41541-020-00240-0 (PMC7550607; doi:10.1038/s41541-020-00240-0)
Supplement: Supplementary file 1 — Supplementary Information [file 41541_2020_240_MOESM1_ESM.pdf]

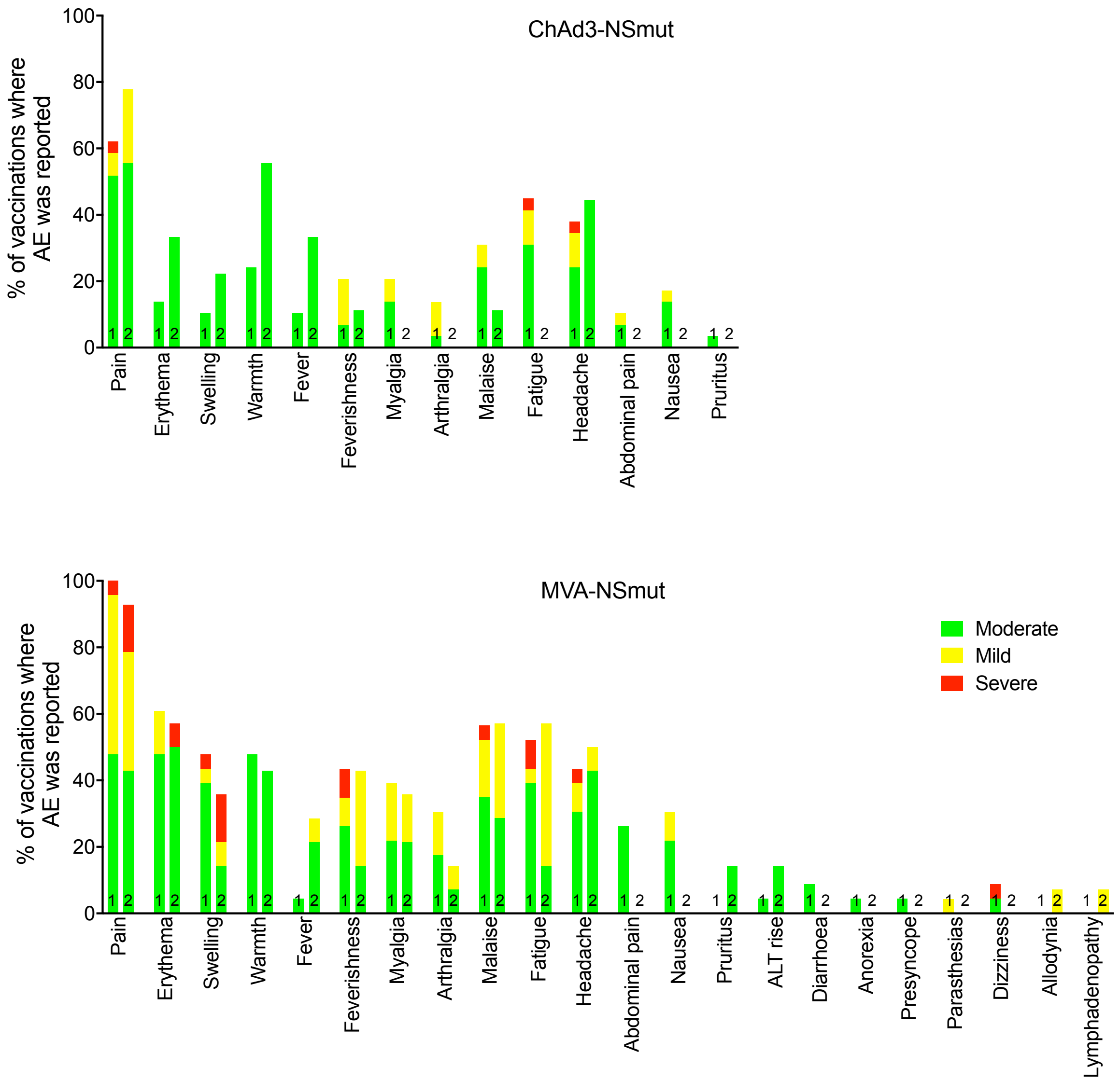

Supplementary Figure 1: Reboosting with ChAd3-NSmut and MVA-NSmut is safe and well tolerated. The percentage of volunteers reporting an adverse event following 1st (1) or 2nd (2) vaccination with ChAd3-NSmut (top) or MVA-NSmut (bottom). Severity moderate (grade 1, green), mild (grade 2, yellow), and severe (grade 3, red).

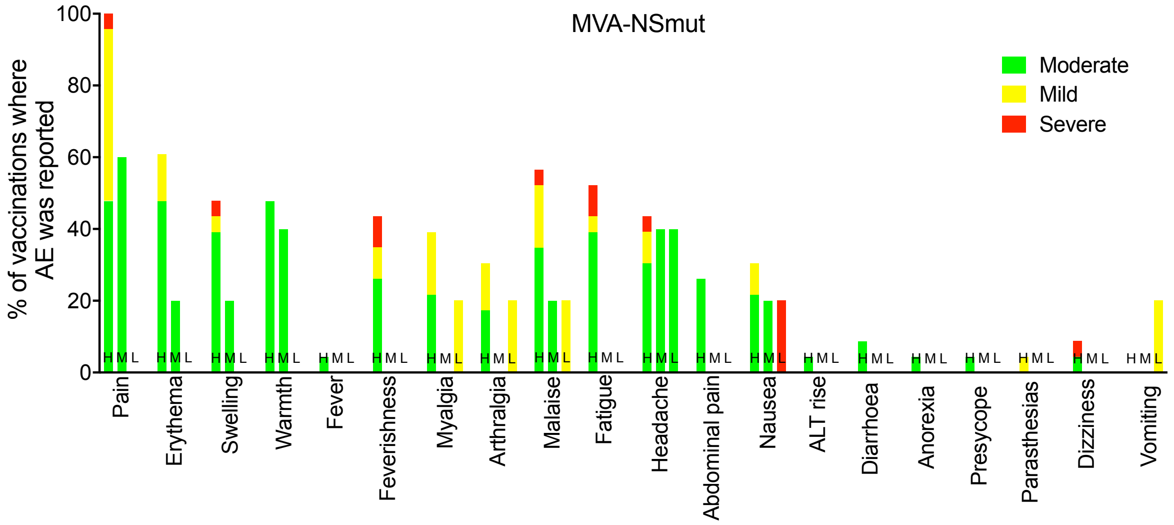

Supplementary Figure 2: Reactogenicity of high, medium and low dose MVA-NSmut in healthy volunteers. Adverse events following high **(H; 10^8 pfu)** medium **(M; 10^7 pfu**) and low dose **(L; 10^6 pfu**) vaccinations with MVA-NSmut. The percentage of vaccinations resulting in each adverse event. Severity moderate (grade 1, green), mild (grade 2, yellow), and severe (grade 3, red).

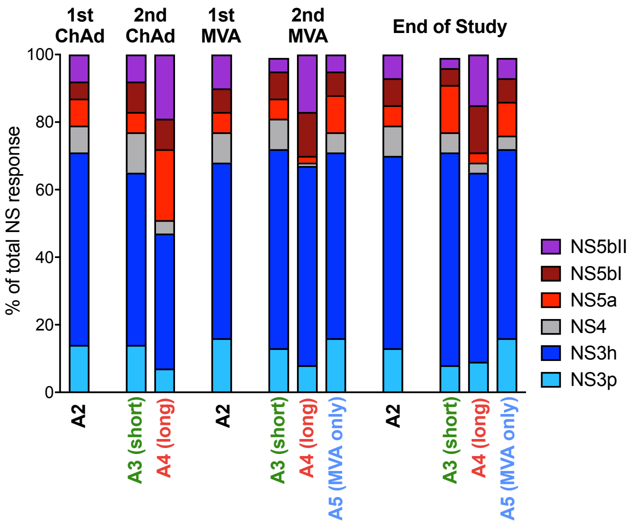

Supplementary Figure 3: Immunodominance of HCV non-structural proteins encoded within the vaccine transgene after reboosting vaccinations: The percentage contribution of the T cell response to HCV non-structural (NS) proteins to the total HCV NS T cell response by IFNγ ELISpot at the peak of the T cell response post-vaccination and at the end of the study (2-4wks post ChAd3-NSmut and 1-4wks post-MVA-NSmut for peak response and 14-40wks post vaccination for end of study; group mean per peptide pool, n = 4-9).

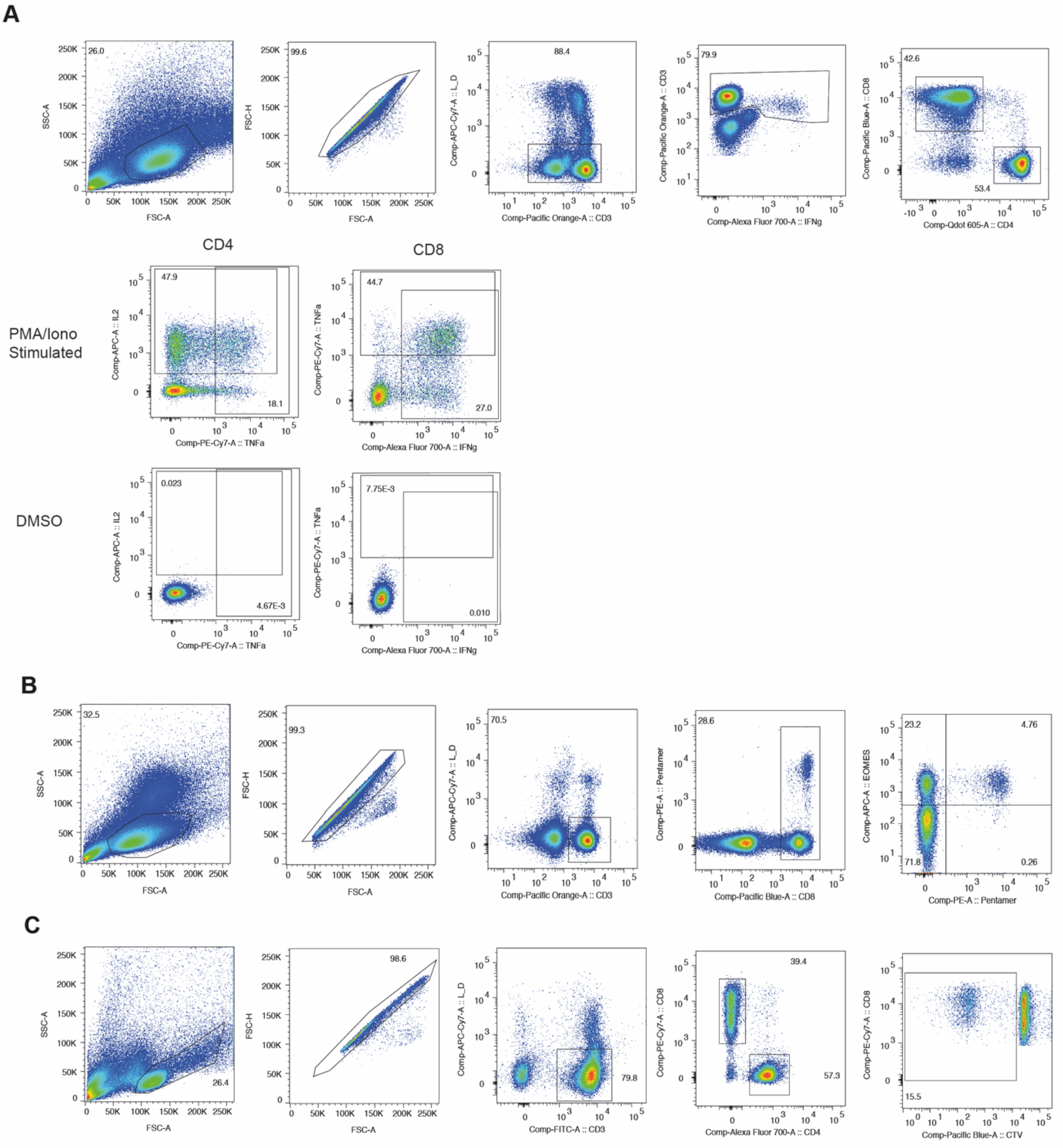

Supplementary Figure 4: Flow cytometry gating strategies. The gating strategy to sequentially identifying lymphocytes, singlets, live cells, and CD3$^+$ CD4$^+$ or CD8$^+$ T cells are shown for intracellular cytokine staining **(A; see Figure 3, 7)**, MHC class I pentamer phenotypic staining (surface and intranuclear) **(B; see Figure 4, 6, 7)** and cell trace violet proliferation assay **(C; see Figure 8)**. **(A)** Also shown are examples plots of IL-2 vs. IFNγ or TNFα vs. IFNγ are shown for Phorbol 12-Myristate 13-Acetate (PMA)/Ionomycin stimulated or DMSO unstimulated wells gated on CD4$^+$ (left) or CD8$^+$ T cells (right). **(B)** MHC Class I Pentamer vs. intranuclear stained EOMES. **(C)** Also shown, CD8 vs. Cell Trace Violet (CTV) is shown for PBMC stimulated with HCV NS3h.

Trial Arm	n	Vaccine 1 (TW0)	Vaccine 2 (TW8)	Vaccine 3	Vaccine 4
A1 *	4	**MVA**			
A2 *	9	ChAd3	**MVA**		
A3	5	ChAd3	**MVA**	ChAd3 (TW16)	**MVA** (TW24)
A4	4	ChAd3	**MVA**	ChAd3 (TW47-92)	**MVA** (TW55-100)
A5	5	ChAd3	**MVA**	**MVA** (TW40)	
A6	5	ChAd3	**MVA** (2 x 10^7 pfu)		
A7	5	ChAd3	**MVA** (2 x 10^6 pfu)		

Supplementary Table 1: Study arms, vaccinations given, and doses: Vaccine dose 2.5 x 10^{10} viral particles (vp) for ChAd3-NSmut and 2 x 10^8 plaque forming units (pfu) for MVA-NSmut unless otherwise stated. ChAd3, chimpanzee-derived adenovirus 3; MVA, modified vaccinia Ankara; n, number of individuals in trial arm; TW , Trial week at which vaccine was given. * previously published in Swadling et al 2014.

Trial Arm	Volunteer	Arm	Age (yrs)	Sex	HLA-A	HLA-B	HLA-C	HLA-DRB1	HLA-DRB3/4	HLA-DQ1
A3: ChAd3/MVA/ChAd3/MVA	**318**	A3	25	F	1 & 68	51 & 37	6 & 5	10 & 13	52	5 & 6
	**327**	A3	22	F	24 & 2	44 & 60	2 & 10	17 & 7	52 & 53	2 & 9
	**328**	A3	27	M	2 & 11	62 & 35	9 & 4	14 & 13	52	5 & 6
	**331**	A3	27	F	2 & 68	65 & 27	8 & 1	4 & 13	52 & 53	7 & 6
	**332**	A3	21	M	3 & 2	44 & 62	5 & 10	4	53	8 & 7
A4: ChAd3/MVA_ChAd3/MVA	**333**	A4	21	F	11	39 & 57	12 & 6	1 & 7	53	9 & 5
	**334**	A4	21	F	2 & 1	8 & 44	5 & 7	11 & 17	52 & 53	2 & 7
	**335**	A4	22	F	2 & 31	27 & 7	7 & 2	4 & 15	51 & 53	6 & 8
	**337**	A4	19	M	2	13 & 57	6	7	53	2 & 9
A5: ChAd3/MVA_MVA	**338**	A5	22	F	3 & 24	44 & 65	8 & 16	7 & 13	52 & 53	2 & 6
	**339**	A5	37	F	1 & 2	8 & 60	7 & 10	4 & 14	52 & 53	5 & 8
	**341**	A5	41	M	1 & 3	18 & 65	7 & 8	11 & 13	52	6 & 7
	**343**	A5	30	M	2	27 & 49	1 & 7	1	-	5
	**345**	A5	21	F	2 & 32	44	5	4 & 15	51 & 53	6 & 7
A6: ChAd3/MVA (Medium dose, 2×10^7 pfu)	**346**	A6	33	M	3 & 68	7 & 58	7	13 & 16	51 & 52	5 & 6
	**347**	A6	26	M	2 & 11	7	7	15 & 103	51	5 & 6
	**348**	A6	26	F	3 & 32	8 & 27	2 & 7	14 & 17	52	2 & 5
	**349**	A6	20	F	2 & 24	27 & 65	7 & 8	103 & 7	53	2 & 7
	**368**	A6	22	M	24	44 & 51	5 & 15	4 & 13	52 & 53	7
A7: ChAd3/MVA (Low dose, 2×10^6 pfu)	**370**	A7	22	M	26 & 29	44 & 45	6 & 16	1 & 7	53	2 & 5
	**371**	A7	22	F	32 & 68	27 & 51	1 & 14	7 & 8	53	2 & 4
	**372**	A7	20	F	1 & 24	27 & 57	2 & 6	4 & 7	53	7 & 9
	**373**	A7	22	M	1 & 3	8 & 51	1 & 7	1 & 13	52 & 53	5 & 6
	**374**	A7	28	F	2 & 3	7 & 62	7 & 10	4 & 15	51 & 53	6 & 8

Supplementary Table 2: Volunteer demographics: Age, Age at time of vaccination; ChAd, chimpanzee derived adenovirus; F, female; HLA, human leukocyte antigen; M, male; MVA, modified vaccinia Ankara; Pfu, plaque forming units.

Arm(n)	1st ChAd3-NSmut	n=29	
	mild	moderate	severe
A2 (9)	23	9	3
A3 (5)	19	2	0
A5 (5)	8	3	0
A6 (5)	15	0	0
A7 (5)	4	7	0
AE per vaccination	2.4	0.7	0.1
	2nd ChAd3-NSmut	n=9	
A3 2nd (5)	11	0	0
A4 2nd (4)	13	2	0
AE per vaccination	2.7	0.2	0.0

Arm (n)	1st MVA-NSmut	n = 23	
	mild	moderate	severe
A1 (4)	26	4	3
A2 (9)	39	9	6
A3 (5)	16	12	0
A5 (5)	18	9	0
AE per vaccination	4.3	1.5	0.4
	2nd MVA-NSmut	n = 14	
A3 2nd (5)	20	5	1
A4 2nd (4)	15	11	2
A5 2nd (5)	11	11	2
AE per vaccination	3.3	1.9	0.4

Supplementary Table 3: The number of adverse events for each vaccination by severity: AE, adverse event; ChAd, chimpanzee-derived adenovirus; MVA, modified vaccinia Ankara. n = number of individuals vaccinated.

Arm (n)			
High dose MVA-NSmut (10^8 pfu)	mild	moderate	severe
A1 (4)	26	4	3
A2 (9)	39	9	6
A3 (5)	16	12	0
A5 (5)	18	9	0
AE per vaccination (n=23)	4.3	1.5	0.4
	mild	moderate	severe
Medium dose MVA-NSmut 10^7 pfu (A6)	11	0	0
AE per vaccination (n=5)	2.2	0	0
Low dose MVA-NSmut 10^6 pfu (A7)	2	4	1
AE per vaccination (n=5)	0.4	0.8	0.2

Supplementary Table 4: The number of adverse events by severity for high, medium, and low dose MVA-NSmut vaccination: AE, adverse event; ChAd, chimpanzee-derived adenovirus; MVA, modified vaccinia Ankara. n = number of individuals vaccinated.
