# Supplementary material for: Optimising T cell (re)boosting strategies for adenoviral and modified vaccinia Ankara vaccine regimens in humans
Source: NPJ Vaccines. 2020 Oct 12;5:94. doi: 10.1038/s41541-020-00240-0 (PMC7550607; doi:10.1038/s41541-020-00240-0)
Supplement: Supplementary file 2 — Reporting Summary [file 41541_2020_240_MOESM2_ESM.pdf]

nature research

Corresponding author(s): Leo Swadling, Eleanor Barnes

Last updated by author(s): 08/18/2020

Reporting Summary

Nature Research wishes to improve the reproducibility of the work that we publish. This form provides structure for consistency and transparency in reporting. For further information on Nature Research policies, see our Editorial Policies and the Editorial Policy Checklist.

Statistics

For all statistical analyses, confirm that the following items are present in the figure legend, table legend, main text, or Methods section.

n/a	Confirmed	
☐	☒	The exact sample size (n) for each experimental group/condition, given as a discrete number and unit of measurement
☐	☒	A statement on whether measurements were taken from distinct samples or whether the same sample was measured repeatedly
☐	☒	The statistical test(s) used AND whether they are one- or two-sided<br>*Only common tests should be described solely by name; describe more complex techniques in the Methods section.*
☒	☐	A description of all covariates tested
☐	☒	A description of any assumptions or corrections, such as tests of normality and adjustment for multiple comparisons
☐	☒	A full description of the statistical parameters including central tendency (e.g. means) or other basic estimates (e.g. regression coefficient) AND variation (e.g. standard deviation) or associated estimates of uncertainty (e.g. confidence intervals)
☒	☐	For null hypothesis testing, the test statistic (e.g. F, t, r) with confidence intervals, effect sizes, degrees of freedom and P value noted<br>*Give P values as exact values whenever suitable.*
☒	☐	For Bayesian analysis, information on the choice of priors and Markov chain Monte Carlo settings
☒	☐	For hierarchical and complex designs, identification of the appropriate level for tests and full reporting of outcomes
☐	☒	Estimates of effect sizes (e.g. Cohen's d, Pearson's r), indicating how they were calculated

Our web collection on statistics for biologists contains articles on many of the points above.

Software and code

Policy information about availability of computer code

Data collection	No software was used for data collection
Data analysis	Graphpad Prism v. 7.0e for Mac was used for statistical analysis. FlowJo (Tree Star) version 10.4.1 was used for flow cytometry analysis. Pestle version 1.8 and Spice version 6.0 were used for background subtraction, data formatting, and data visualistion for polyfunctionality assessment of ICS data.

For manuscripts utilizing custom algorithms or software that are central to the research but not yet described in published literature, software must be made available to editors and reviewers. We strongly encourage code deposition in a community repository (e.g. GitHub). See the Nature Research guidelines for submitting code & software for further information.

Data

Policy information about availability of data

All manuscripts must include a data availability statement. This statement should provide the following information, where applicable:

- Accession codes, unique identifiers, or web links for publicly available datasets
- A list of figures that have associated raw data
- A description of any restrictions on data availability

The data that support the findings of this study are available from the corresponding author L.S upon reasonable request. This study did not generate any unique code or datasets other than those included in this published article (and its supplementary information files).

Field-specific reporting

Please select the one below that is the best fit for your research. If you are not sure, read the appropriate sections before making your selection.

☒ Life sciences ☐ Behavioural & social sciences ☐ Ecological, evolutionary & environmental sciences

For a reference copy of the document with all sections, see nature.com/documents/nr-reporting-summary-flat.pdf

Life sciences study design

All studies must disclose on these points even when the disclosure is negative.

Sample size	No sample size calculation was performed for this first-in-man phase I study. Group sizes of 4-10 healthy volunteers per trial arm are standard for phase I studies of safety and immunogenicity.
Data exclusions	No data were excluded from analysis.
Replication	All ELISpot data is performed in triplicate and assay robustness and reproducibility of this assay and ICS assay have been presented in previous publications Barnes et al STM 2012, Swadling et al 2014.
Randomization	For this first-in-man phase I study no randomisation was used as stated in the manuscript.
Blinding	For this first-in-man phase I study no blinding was used as stated in the manuscript.

Reporting for specific materials, systems and methods

We require information from authors about some types of materials, experimental systems and methods used in many studies. Here, indicate whether each material, system or method listed is relevant to your study. If you are not sure if a list item applies to your research, read the appropriate section before selecting a response.

Materials & experimental systems

n/a	Involved in the study	
☐	☒	Antibodies
☒	☐	Eukaryotic cell lines
☒	☐	Palaeontology and archaeology
☒	☐	Animals and other organisms
☐	☒	Human research participants
☐	☒	Clinical data
☒	☐	Dual use research of concern

Methods

n/a	Involved in the study	
☒	☐	ChIP-seq
☐	☒	Flow cytometry
☒	☐	MRI-based neuroimaging

Antibodies

Antibodies used	The fluorochrome-antibody combination and manufacturer for all FACS antibodies are given in the materials and methods.
Validation	Example gating of all antibodies and MHC Class I pentamers are given alongside the summary data in main figures of the manuscript and full gating panels are provided in supplementary figure 4.

Human research participants

Policy information about studies involving human research participants

Population characteristics	Volunteer covariate-relevant characteristics Sex, Age, and MHC class I HLA typing are provided for all participants.
Recruitment	Volunteers were recruited at the CCVTM (Centre for Clinical Vaccinology and Tropical Medicine), Churchill Hospital, Oxford.
Ethics oversight	Approvals for the study were granted by the UK National Research Ethics Service, (NRES Committee South Central – Oxford REC A, REC reference number 10/H0604/45) and the UK Medicines and Healthcare Products Regulatory Agency (EudraCT no. 2009-018260-10). GCP compliance was independently monitored by the University of Oxford Clinical Trials and Research Governance office. A multinational independent data safety monitoring committee (DSMC) provided safety oversight.

Note that full information on the approval of the study protocol must also be provided in the manuscript.

Clinical data

Policy information about clinical studies

All manuscripts should comply with the ICMJE guidelines for publication of clinical research and a completed CONSORT checklist must be included with all submissions.

Clinical trial registration	ClinicalTrial.gov database (ID: NCT01296451, December 2010)
Study protocol	ClinicalTrial.gov database (ID: NCT01296451)
Data collection	Volunteers were recruited at the CCVTM (Centre for Clinical Vaccinology and Tropical Medicine), Churchill Hospital, Oxford. Recruitment started Dec 2010 and the primary completion data of the study was Jan 2016.
Outcomes	Primary outcome measures:<br>Number of participant with adverse events, type and severity of adverse events [Time Frame: Different time frames depending on study groups] To assess the safety of new hepatitis C vaccine candidates, AdCh3NSmut (and the improved version AdCh3NSmut1) and MVA-NSmut when administered to healthy volunteers and to HCV infected patients. The specific endpoints for safety were active collection of data on adverse events.<br><br>Secondary Outcome Measures :<br>Immunogenicity [Time Frame: Different time frames depending on the study groups]<br>To assess the cellular immune response generated by AdCh3NSmut (and the improved version AdCh3NSmut1) and MVA-NSmut, when administered sequentially, to patients with hepatitis C virus infection. The specific endpoint of cellular immune response was collected via IFN-gamma ELISpot assay and other exploratory immunological tests.

Flow Cytometry

Plots

Confirm that:

☒ The axis labels state the marker and fluorochrome used (e.g. CD4-FITC).

☒ The axis scales are clearly visible. Include numbers along axes only for bottom left plot of group (a 'group' is an analysis of identical markers).

☒ All plots are contour plots with outliers or pseudocolor plots.

☒ A numerical value for number of cells or percentage (with statistics) is provided.

Methodology

Sample preparation	PBMC were separated via density gradient (Lymphoprep) and counted using a Guava Personal Cell Analyser (Merck Millipore).
Instrument	BD LSRII flow cytometer
Software	FlowJo (Tree Star) version 10.4.1
Cell population abundance	No FACS sorting was performed
Gating strategy	FACS gating strategies are described in figure legends for all panels including FACS data and example plots of each fluorescent antibody and MHC class I pentamers are shown in figures. As stated in the materials and methods section, Fluorescence minus one controls are used where relevant, in particular for all phenotyping markers. Unstimulated wells are run in parallel for each sample (volunteer and time point) for ICS which are used to define cytokine gates and for background subtraction. Unstimulated and unstained controls were ran in parallel (examples shown; for a given sample - volunteer and time point specific) for all CTV stains.

☒ Tick this box to confirm that a figure exemplifying the gating strategy is provided in the Supplementary Information.
